# A Cell-Penetrating Peptide with a Guanidinylethyl Amine Structure Directed to Gene Delivery

**DOI:** 10.1038/srep19913

**Published:** 2016-01-27

**Authors:** Makoto Oba, Takuma Kato, Kaori Furukawa, Masakazu Tanaka

**Affiliations:** 1 Graduate School of Biomedical Sciences, Nagasaki University, 1-14 Bunkyo-machi, Nagasaki 852-8521, Japan

## Abstract

A peptide composed of lysine with a guanidinylethyl (GEt) amine structure in the side chain [Lys(GEt)] was developed as a cell-penetrating peptide directed to plasmid DNA (pDNA) delivery. The GEt amine adopted a diprotonated form at neutral pH, which may have led to the more efficient cellular uptake of a Lys(GEt)-peptide than an arginine-peptide at a low concentration. Lys(GEt)-peptide/pDNA complexes showed the highest transfection efficiency due to efficient endosomal escape without any cytotoxicity. Lys(GEt)-peptide may be a promising candidate as a gene delivery carrier.

Cell-penetrating peptides (CPPs) are one of the powerful tools used to deliver drugs, proteins, and nucleic acids^1^,^2^. The mixing of cargo with CPPs allows for its effective introduction into a cell^3^. CPPs have also been used for the high functionalization of drug delivery systems such as liposomes and polymeric micelles by chemical modifications^4^,^5^,^6^. Novel CPPs are expected to be developed with excellent cell-penetrating abilities and no cytotoxicity. Arginine (Arg)-rich peptides have been identified as some of the most efficient CPPs^7^,^8^,^9^,^10^,^11^,^12^. Cationic guanidino groups in the side chain of Arg are critical for cell penetration. Therefore, novel CPPs have been developed based on Arg-rich peptides and their derivatives^13^,^14^,^15^,^16^,^17^.

Polycations containing ethylenediamine structures, as represented by polyethyleneimine^18^,^19^,^20^, are well-known efficient gene delivery carriers, and many studies have been devoted to increasing the transfection efficiencies (TE) and lowering the cytotoxicities of these carriers^21^,^22^,^23^,^24^. The mechanisms responsible for efficient transfection by polycations with ethylenediamine have recently been elucidated in detail^25^,^26^,^27^. The degree of protonation plays a crucial role in high endosomal-escaping abilities. The membrane-destabilizing capacity of a monoprotonated gauche form at neutral pH was previously shown to be low, whereas that of a diprotonated anti-form at acidic pH was high, resulting in high endosomal escape with negligible cytotoxicity. Diprotonated ethylenediamine structures with higher cationic charge densities have the potential to associate with the cell membrane and deliver cargo into a cell.

In the present study, we designed a CPP that was equipped with the properties of a diprotonated ethylenediamine and Arg-rich peptide for the purpose of plasmid DNA (pDNA) delivery. The unnatural amino acid described herein was a lysine (Lys) derivative with a guanidinylethyl (GEt) group in the side chain amine [Lys(GEt)] (Fig. 1). The pKa of a protonated guanidine (pKa 12.5 for Arg) is known to be higher than that of a protonated primary amine (pKa 10.2 for Lys)^28^. The di-guanidinylation of primary amines in a diethylenetriamine was previously reported to shift the pKa of its protonated secondary amine from 3.9 to 6.3^29^. Cyclodextrins^30^ and block polymers^31^ with a GEt amine structure have been shown to have high cell-penetrating abilities and *in vitro* gene transfer, respectively. We assumed that the side chain of Lys(GEt) adopted the diprotonated form at neutral pH and, therefore, its oligopeptide may exhibit a high cell-penetrating ability and effective endosomal escape, even at physiological pH, ultimately resulting in efficient transfection efficiency. The oligopeptides of Lys, Arg, and Lys(AEt), which has an aminoethyl (AEt) group in the side chain amine of Lys, were also prepared as controls (Fig. 1), and all of the peptides prepared were evaluated for their cell-penetrating abilities and pDNA delivery.

## Results and Discussion

### Preparation of peptides

**Figure 1:**
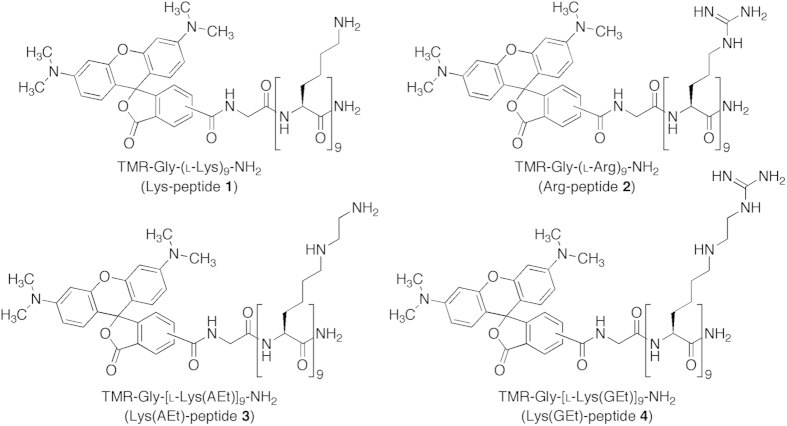
Structures of cell-penetrating peptides **1**–**4** designed in the present study.

The N-terminal-protected amino acids, Fmoc-l-Lys[Boc,AEt(Boc)]-OH and Fmoc-l-Lys[Boc,GEt(Boc)_2_]-OH, were synthesized according to Supplementary Scheme S1 and Scheme S2, respectively. Tetramethylrhodamine carboxylic acid (TMR)-labeled peptides **1**–**4** (Fig. 1) were prepared by the Fmoc solid-phase method using (1-cyano-2-ethoxy-2-oxoethylidenaminooxy)dimethylamino-morpholino-carbenium as a coupling reagent (Supplementary Information). TMR was introduced for the fluorescent label in order to evaluate the cell-penetrating ability and intracellular distribution of each peptide. The peptides were then purified with reverse-phase HPLC. The homogeneities and purities of the peptides were verified by analytical reverse-phase HPLC and matrix-assisted laser desorption-ionization time-of-flight mass spectrometry (Supplementary Fig. S1, S2). Carboxyfluorescein (CF)-labeled Arg-peptide **5** was prepared in the same manner as TMR-labeled Arg-peptide **2** and as described previously^32^,^33^.

### Potentiometric titration of model compounds

**Figure 2:**
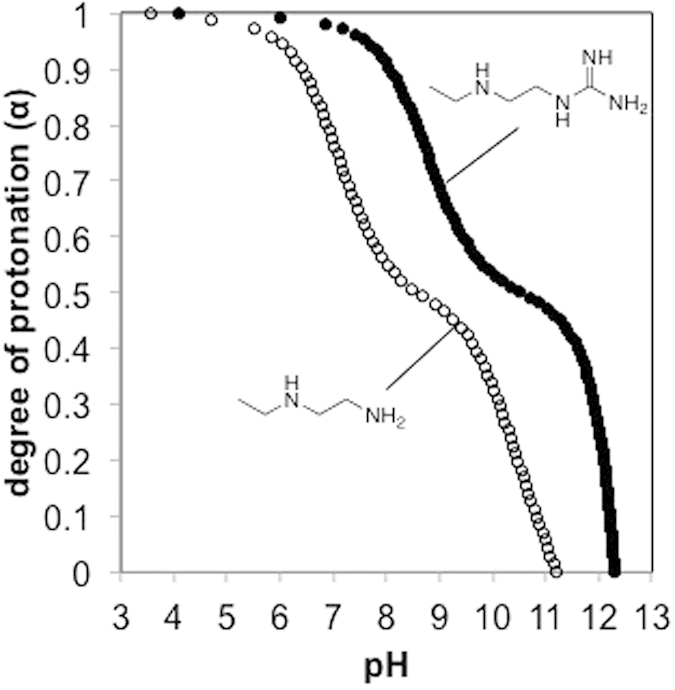
α/pH curves of model compounds for Lys(AEt) (open circles) and Lys(GEt) (filled circles).

1-Ethylamino-2-guanidinylethane hydrochloride, a model compound for Lys(GEt), was synthesized according to Supplementary Scheme S3 in order to clarify the protonation degree of the GEt amine structure within a range from pH 3 to pH 13. *N*-Ethylethylenediamine hydrochloride, a model compound for Lys(AEt), exhibited a distinctive two-step protonation behavior (α = 0.97 at pH 5.5 and α = 0.66 at pH 7.4; pK_a1_ 7.1 and pK_a2_ 10.3) (Fig. 2), which was consistent with previous findings on polycations with ethylenediamine^25^,^26^,^27^. The monoprotonate state was a major form of the AEt amine structure at neutral pH. On the other hand, the model compound for Lys(GEt) showed a similar distinctive two-step protonation behavior with a shift to a high pH (α = 0.99 at pH 5.5 and α = 0.96 at pH 7.4; pK_a1_ 8.8 and pK_a2_ 12.0) (Fig. 2). The GEt amine structure took the diprotonated form even at neutral pH, as expected. The primary amine and guanidine were considered to be α > 0.99 at pH 7.4 based on the pKa of Lys (10.2) and Arg (12.5)^28^.

### Cellular uptake and cell viability using peptides

**Figure 3:**
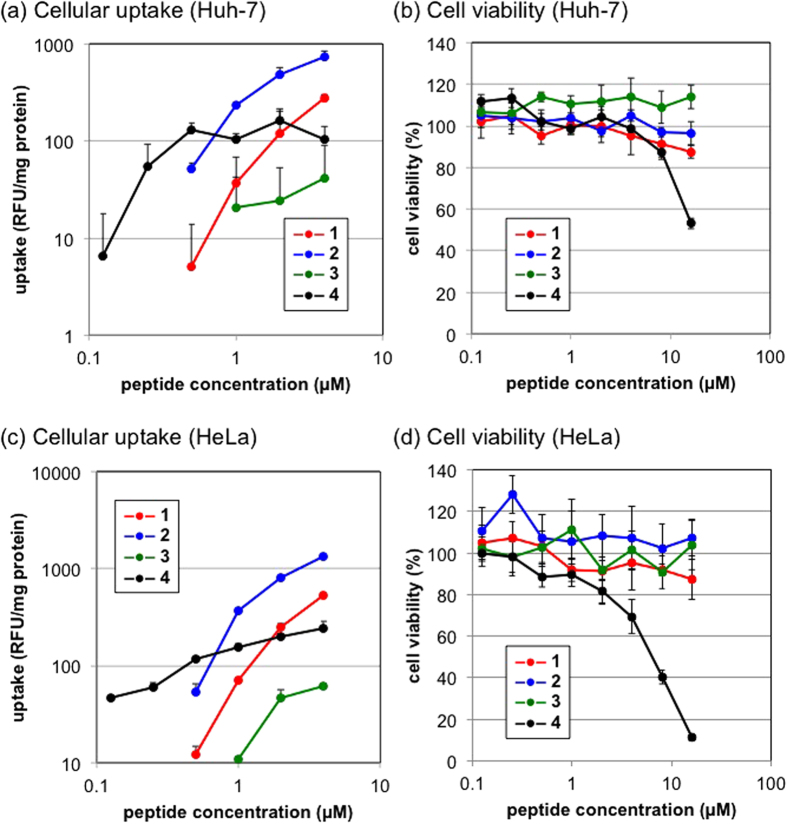
Cellular uptake (**a,c**) and cell viability (**b,d**) of peptides **1**–**4** by Huh-7 cells (**a,b**) and HeLa cells (**c**,**d**). Peptide concentration-dependency with a 2-h incubation period. Error bars represent the standard deviation, n = 3 (**a**) and n = 5 (**b**).

The cellular uptake of peptides **1**–**4** into Huh-7 cells (Fig. 3a) and HeLa cells (Fig. 3c) was evaluated at different concentrations. Arg-peptide **2** showed the most efficient cellular uptake at concentrations not less than 1 μM. On the other hand, at low concentrations of 0.5, 0.25, and 0.125 μM, the cellular uptake of Lys(GEt)-peptide **4** was significantly higher than that of the other peptides examined (e.g., *P* < 0.05, peptide **2** vs peptide **4**, peptide concentration: 0.5 μM, Fig. 3a). The cellular uptake of peptides **1**, **2**, and **3** was undetectable at concentrations of less than 0.5, 0.5, and 1 μM, respectively. A cell viability assay revealed the moderate cyototoxicity of Lys(GEt)-peptide **4** at a high concentration (Fig. 3b,d). The diprotonated GEt amine (Lys(GEt)-peptide **4**) appeared to associate more strongly with the cell membrane than the protonated primary amine (Lys-peptide **1**), protonated guanidine (Arg-peptide **2**), and monoprotonated ethylenediamine (Lys(AEt)-peptide **3**), which may have led to its efficient intracellular internalization at a low concentration and enhanced cyototoxicity at a high concentration. The rank order of cellular uptake at a peptide concentration of 1 μM was the same at all incubation times (Supplementary Fig. S3).

### CLSM observations of peptides

**Figure 4:**
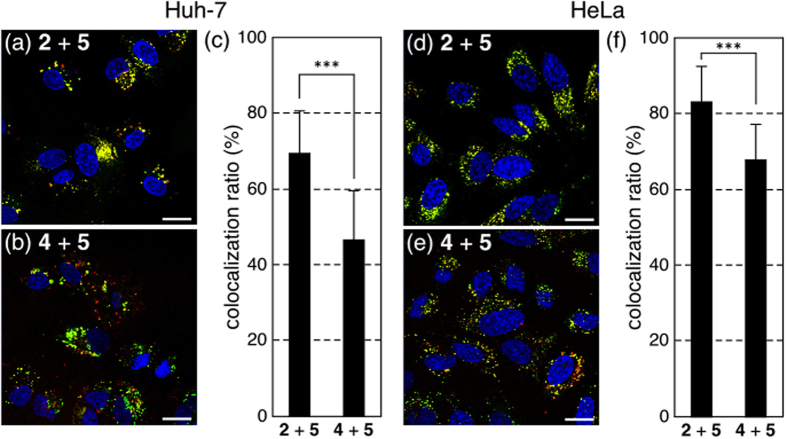
Intracellular distributions of TMR-labeled Arg-peptide **2** (**a,d**) and Lys(GEt)-peptide **4** (**b,e**) with CF-labeled Arg-peptide **5**. Peptides **2** or **4** (red) and peptide **5** (green) were simultaneously added and incubated with Huh-7 cells (**a–c**) or HeLa cells (**d–f**) for 2 h. CLSM observations were carried out after staining nuclei with Hoechst 33342 (blue). The scale bars represent 20 μm. (**c**,**f**) Quantification of peptides **2** and **4** colocalized with peptide **5**. The error bars represent the standard deviation, n = 20. ***P < 0.001.

The intracellular distributions of Arg-peptide **2** and Lys(GEt)-peptide **4** was investigated using CLSM in order to gain insights into their different intracellular distributions. CLSM observations of Huh-7 cells or HeLa cells treated with TMR-labeled Arg-peptide **2** or Lys(GEt)-peptide **4** together with CF-labeled Arg-peptide **5** provided direct information on their different intracellular distributions (Fig. 4). Cells treated with Arg-peptides **2** (red) and **5** (green) were expected to show the same distribution (yellow) because the same peptide sequence was used. The spots observed in cells treated with peptides **2** and **5** were mainly yellow spots (Fig. 4a,d), whereas some of the fraction was observed as red and green spots in cells treated with peptides **4** and **5** (Fig. 4b,e). The rate of colocalization was quantified, and 70% of Arg-peptide **2** and 47% of Lys(GEt)-peptide **4** colocalized with Arg-peptide **5** in Huh-7 cells (Fig. 4c). A significant difference was observed between peptides **2** and **4** (*P* < 0.001), and, therefore, the final destinations of peptides **2** and **4** in Huh-7 cells appeared to be different. HeLa cells treated with peptides **2** and **4** also showed colocalization ratios of 83% and 67% with peptide **5**, respectively (*P* < 0.001) (Fig. 4f). Lys(GEt)-peptide **4** was more strongly associated with the cell membrane and internalized into cells more efficiently at a low concentration by a slightly different mechanism than that of Arg-peptide **2**.

### Preparation and characterization of peptide/pDNA complexes

**Figure 5:**
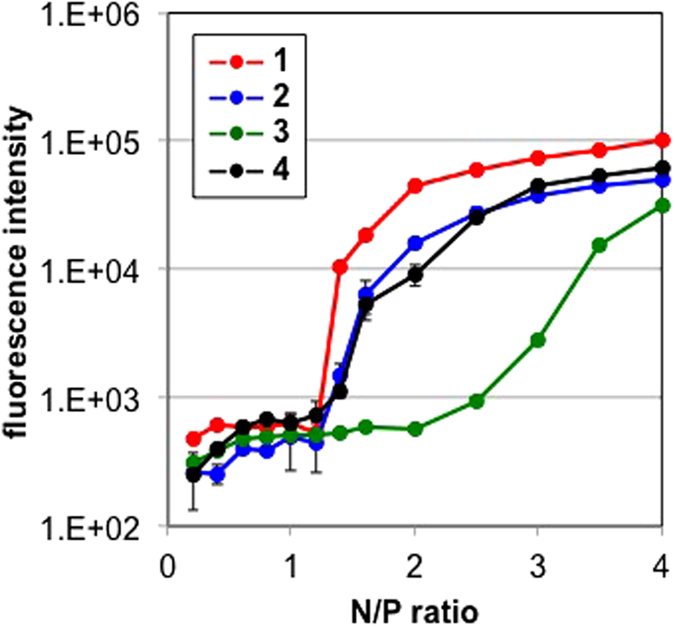
Fluorescence intensities of peptide/pDNA complex solutions at various N/P ratios. Error bars represent the standard deviation, n = 3.

We then evaluated peptide/pDNA complexes for gene delivery. The formation of peptide/pDNA complexes was confirmed by fluorescence measurements of TMR-labeled peptide/pDNA complex solutions prepared at various N/P ratios, which were defined as the residual molar ratio of the amino and/or guanidino groups of amino acids in the peptide to the phosphate groups of pDNA (Fig. 5). The model compounds for Lys(AEt) and Lys(GEt) exhibited α = 0.66 and 0.96 at pH 7.4 (Fig. 2), and thus, an N/P ratio = 2 means that 1.32 and 1.94 of the residual molar ratio of the protonated amino and/or guanidino groups to the phosphate groups, respectively. Fluorescence quenching was expected to occur with the formation of assembly structures through self-quenching^12^,^34^,^35^. Peptides **1**, **2**, and **4** showed similar curves, in which fluorescence intensities were elevated between N/P ratios 1.2 and 1.4, suggesting a stoichiometric N/P ratio in these complexes. On the other hand, the fluorescence intensity of Lys(AEt)-peptide **3** gradually increased from an N/P ratio = 2, which was consistent with previous findings in which all pDNA were associated with polycations with ethylenediamine at an N/P ratio = 2^25^,^26^,^27^. The mean size and zeta-potential of the peptide/pDNA complexes were similar among peptides **1**, **2**, and **4** (Supplementary Table S1). At N/P ratios ≥4, their size and zeta-potential were maintained at < 105 nm with a moderate polydispersity index (PDI) and >+14 mV, respectively. The zeta-potential of Lys(AEt)-peptide **3** complexes was still a negative value at an N/P ratio = 2 and their size was bigger than that of the other three complexes, which may have been due to the monoprotonated form of ethylenediamine at neutral pH. DLS measurements of each peptide solution without pDNA showed no informative data (data not shown), and, thus, the peptides themselves did not aggregate.

### Transfection efficiency and cell viability

**Figure 6:**
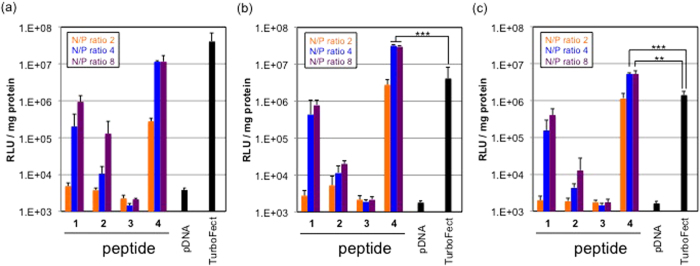
Transfection efficiency of peptide/pDNA complexes with 24-h (a), 48-h (**b**), and 72-h (**c**) post-incubation against Huh-7 cells. The error bars represent the standard deviation, n = 4. **P < 0.01 and ***P < 0.001.

**Figure 7:**
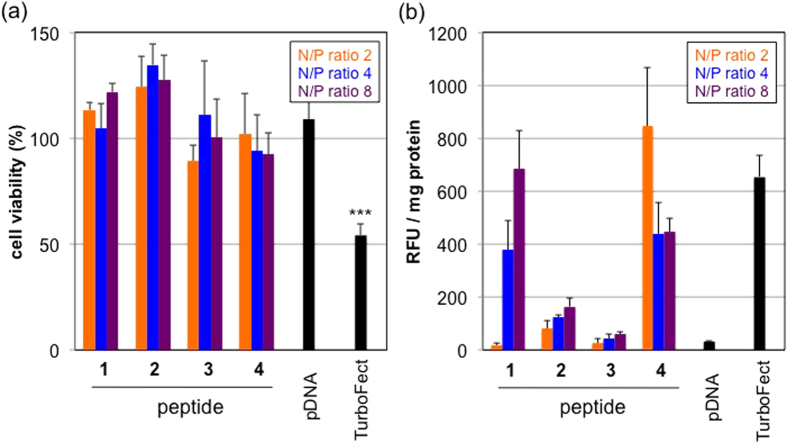
Cell viability (a), and cellular uptake (b) of peptide/pDNA complexes against Huh-7 cells. The error bars represent the standard deviation, n = 4 (**a**) and n = 3 (**b**). ***P < 0.001, vs control.

The TE of the pDNA encoding luciferase against Huh-7 cells was compared among peptide/pDNA complexes with naked pDNA as a negative control and commercially available reagent TurboFect as a positive control (Fig. 6). The TE of all the peptides increased with increasing N/P ratios, probably due to the effect of unbound free peptides^36^,^37^. The amount of free peptides was different in each peptide, which might be one of the reasons for different TE of each peptide. Lys(GEt)-peptide **4** at N/P ratios = 4 and 8 showed the highest TE of all the peptides examined and was higher than TurboFect 48-h and 72-h post-incubation (Fig. 6b,c). The post-incubation time affected the transfection ability of each complex. Lys-peptide **1**, Arg-peptide **2**, and TurboFect showed the maximum TE approximately 24-h post-incubation, while Lys(GEt)-peptide **4** showed it at 48-h post-incubation. The TE of TurboFect was significantly higher than that of Lys(GEt)-peptide **4** with a shorter post-incubation (Fig. 6a). On the other hand, with a longer post-incubation (≥48 h), Lys(GEt)-peptide **4** showed the most efficient transfection. Lys(GEt)-peptide **4** appeared to maintain gene expression at a high level for a long time. Taken together with these results, Lys(GEt)-peptide **4** appeared to strongly associate with pDNA due to its high cationic charge density and slowly release pDNA with an increase in the incubation time, which may have led to delayed gene expression. The negligible cytotoxicities of all peptide/pDNA complexes were detected under the conditions examined, whereas TurboFect exhibited approximately 50% cell viability (Fig. 7a). Lys(AEt) and Lys(GEt) residues have two amino and/or guanidino groups and, thus, the concentrations of Lys(AEt)-peptide **3** and Lys(GEt)-peptide **4** were 50% those of Lys-peptide **1** and Arg-peptide **2** in the peptide/pDNA complex solutions prepared at the same N/P ratios.

### Cellular uptake of peptide/Cy5-pDNA complexes

The TE of gene delivery carriers often correlates with their cellular uptake. Thus, we examined the cellular uptake of each complex containing Cy5-pDNA (Fig. 7b). The cellular uptake of Cy5-pDNA by Lys(GEt)-peptide **4** was significantly greater than that by Arg-peptide **2** and Lys(AEt)-peptide **3** at all N/P ratios examined; however, the cellular uptake of Cy5-pDNA did not completely correlate with TE. Despite the significantly higher TE, the cellular uptake of Cy5-pDNA complexes from Lys(GEt)-peptide **4** at N/P ratios = 4 and 8 was less than or equal to those from Lys-peptide **1** (N/P ratios = 4 and 8) and TurboFect. Therefore another reason may exist for the high TE of Lys(GEt)-peptide **4**.

### CLSM observations of peptide/Cy5-pDNA complexes

**Figure 8:**
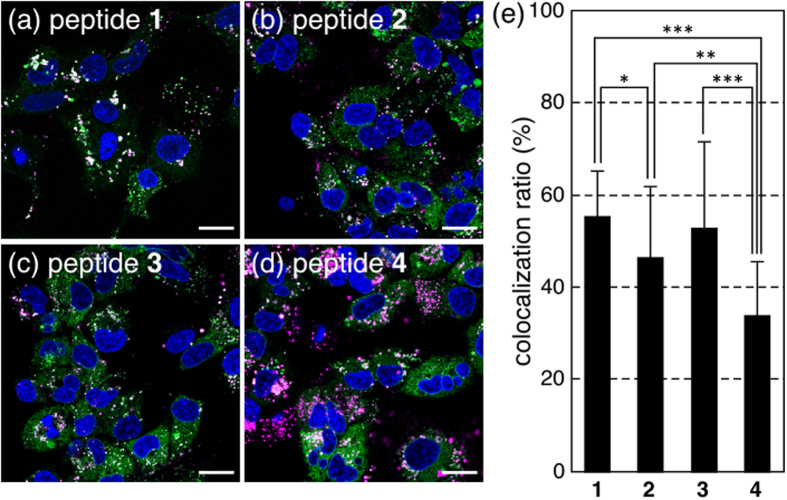
Intracellular distributions of Cy5-pDNA (magenta) with peptides **1** (a), **2** (**b**), **3** (**c**), and **4** (**d**) at an N/P ratio = 4. Acidic late endosomes/lysosomes and nuclei were stained with LysoTracker Green (green) and Hoechst 33342 (blue), respectively. The scale bars represent 20 μm. (**e**) Quantification of the colocalization of Cy5-pDNA with LysoTracker Green. Error bars represent the standard deviation, n = 20. *P < 0.05, **P < 0.01, and ***P < 0.001.

The intracellular distribution of each complex (N/P ratio = 4) containing Cy5-pDNA (magenta) was observed, particularly for endosomal localization, by CLSM after staining late endosomes/lysosomes with LysoTracker Green (green) and nuclei with Hoechst 33342 (blue) (Fig. 8). Efficient endosomal escape is one of the major obstacles for non-viral gene vector, because encapsulated genes are easily degraded by nuclease in lysosome vesicles^38^,^39^. The white pixels represented the colocalization of Cy5-pDNA with LysoTracker Green. Lys(GEt)-peptide **4** complexes appeared to disperse more efficiently in the entire cytoplasmic region than the other complexes. The colocalization ratio of Cy5-pDNA with LysoTracker Green was quantified and shown in Fig. 8e. Approximately 50% of Cy5-pDNA with peptides **1**–**3** was localized in late endosomes/lysosomes, in contrast to only 34% of that with Lys(GEt)-peptide **4**. Lys(GEt)-peptide **4** complexes that internalized into cells achieved effective endosomal escape. Taken together, these results demonstrated that Lys(GEt)-peptide **4** strongly associated with the cell membrane and effectively escaped from endosomes through the synergistic effects of Arg-rich peptides and higher cationic charge densities, which may have led to its superior transfection ability.

In summary, a peptide composed of Lys(GEt) with a GEt amine structure in the side chain was developed as a CPP directed to pDNA delivery. Even at neutral pH, the GEt amine structure adopted the diprotonated form. Lys(GEt)-peptide **4** showed the highest cell-penetrating ability at a low concentration among all the peptides examined, and significant differences were observed in the final cellular destinations of Arg-peptide **2** and Lys(GEt)-peptide **4**. Effective migration of Lys(GEt)-peptide **4**/pDNA complexes from the endosome to the cytoplasm might contribute to their highest TE of all the complexes examined under the same conditions, including a commercially available transfection reagent with negligible cytotoxicity. The Lys(GEt)-peptide is a promising tool for delivering pDNA with minimal cytotoxicity.

## Methods

### Synthesis and characterization of N-terminal-protected amino acids and a model compound

The synthetic schemes of *N*-α-(9-fluorenylmethoxycarbonyl)-*N*-ε-(*tert*-butoxycarbonyl-2′-aminoethyl)-*N*-ε-*tert*-butoxycarbonyl-l-lysine {Fmoc-l-Lys[Boc,AEt(Boc)]-OH}, Fmoc-l-Lys[Boc,GEt(Boc)_2_]-OH, and 1-ethylamino-2-guanidinylethane hydrochloride, a model compound for Lys(GEt), were illustrated in Supplementary Schemes S1, S2, and S3 respectively. Detailed experimental procedures and the characterization of each compound were described in Supplementary data.

### Synthesis and characterization of peptides

Peptides were synthesized on a solid support using Fmoc solid-phase methods with standard commercially available Rink amide resin and Fmoc-amino acids^32^,^33^. Detailed experimental procedures, HPLC charts, and mass spectrometric charts of each peptide were shown in Supplementary data (Supplementary Fig. S1, S2).

### Potentiometric titration

*N*-Ethylethylenediamine hydrochloride (80.5 mg, 0.5 mmol) and 1-ethylamino-2-guanidinylethane hydrochloride (101.5 mg, 0.5 mmol) were separately dissolved in 100 mM HCl (10 mL) to obtain a solution with 100 mM amine and/or guanidine, and then titrated with 100 mM NaOH at 20 °C. Potentiometric titration was performed with a pH meter D-52 (Horiba, Kyoto, Japan). In this experiment, the titrant was added in quantities of 100 μL after the pH values were stabilized.

### Cellular uptake of peptides

Huh-7 cells or HeLa cells were seeded on 24-well culture plates (40000 cells/well) and incubated in 400 μL of DMEM containing 10% fetal bovine serum (FBS). The medium was then replaced with fresh medium containing 10% FBS, and a peptide solution was added to each well at an appropriate concentration (Fig. 3) and at 1 μM (Supplementary Fig. S3). After a 2-h incubation (Fig. 3) and that for each time indicated (Supplementary Fig. S3), the medium was removed and cells were washed with ice-cold PBS and trypsinized. After the addition of medium containing 10% FBS, cells were centrifuged at 1600 rpm for 3 min at 4 °C. The cell pellets obtained were suspended in ice-cold PBS, centrifuged at 1600 rpm for 3 min at 4 °C, and then treated with Cell lysis buffer M. The fluorescence intensity of each lysate was measured using a spectrofluorometer (ND-3300, NanoDrop, Wilmington, DE). The amount of protein in each well was concomitantly determined using the BCA protein assay reagent kit. The results are presented as the mean and standard deviation obtained from 3 samples.

### Cell viability with peptides

Huh-7 cells or HeLa cells were seeded on 96-well culture plates (10000 cells/well) and incubated in 100 μL of DMEM containing 10% FBS. The medium was then replaced with fresh medium containing 10% FBS, and a peptide solution was added to each well at an appropriate concentration. After a 2-h incubation, Cell counting kit-8 was used according to the manufacturer’s protocol. Cell viability was evaluated on the basis of the absorbance of formazan from each well, and 100% cell viability was calculated from the wells without peptides. The results are presented as the mean and standard deviation obtained from 5 samples.

### Confocal laser scanning microscopy (CLSM) observations of peptides 2 and 4 with peptide 5

Huh-7 cells or HeLa cells were seeded onto 8-well chambered cover glasses (Iwaki, Tokyo, Japan) (20000 cells/well) and incubated overnight in 200 μL of DMEM containing 10% FBS. The medium was then replaced with fresh medium containing 10% FBS, and peptide **2** or **4** and peptide **5** were applied to each well at a concentration of 1 μM. After a 2-h incubation, the medium was removed and cells were washed 3 times with ice-cold PBS supplemented with heparin (20 units/mL). The intracellular distribution was observed by CLSM after staining nuclei with Hoechst 33342. CLSM observations were performed using LSM 710 (Carl Zeiss, Oberlochen, Germany) with a Plan-Apochromat 63×/1.4 objective (Carl Zeiss) at an excitation wavelength of 405 nm (UV laser) for Hoechst 33342, 488 nm (Ar laser) for peptide **5**, and 543 nm (He-Ne laser) for peptides **2** and **4**. The rate of the colocalization of peptides **2** and **4** with peptide **5** was quantified^40^,^41^,^42^. The colocalization ratio was quantified as follows:


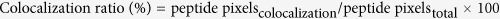


where peptide pixels_colocalization_ represents the number of peptide **2** or **4** pixels colocalizing with peptide **5** in the cell, and peptide pixels_total_ represents the number of all peptide **2** or **4** pixels in the cell. The results are represented as the mean and standard deviation obtained from 20 cells.

### Preparation of peptide/pDNA complexes

Each peptide and pDNA were dissolved separately in 10 mM Hepes buffer (pH 7.3). A two-fold excess volume of peptide solutions of various concentrations was added to the pDNA solution to form peptide/pDNA complexes with different compositions. The final pDNA concentration was adjusted to 33.3 μg/mL and complex solutions were stored at room temperature for 15 min prior to use. The N/P ratio was defined as the residual molar ratio of the amino and/or guanidino groups of amino acids in the peptides to the phosphate groups of pDNA.

### Fluorescent measurements

The fluorescence intensities of peptide/pDNA solutions prepared at various N/P ratios were measured using a spectrofluorometer (ND-3300). Results were presented as the mean and standard deviation obtained from 3 measurements.

### Transfection

Huh-7 cells were seeded on 96-well culture plates (2500 cells/well) and incubated overnight in 100 μL of DMEM containing 10% FBS. The medium was exchanged and peptide/pDNA complex solutions (33.3 μg pDNA/mL) prepared at various N/P ratios were applied to each well (1 μg pDNA/well). After a 24-h incubation, the medium was replaced with 400 μL of fresh medium, followed by each time post-incubation. Luciferase gene expression was then evaluated based on the intensity of photoluminescence using the luciferase assay kit and a luminometer (Gene Light GL-210A, Microtec. Co., Ltd., Chiba, Japan). The amount of protein in each well was concomitantly determined using a Micro BCA protein assay reagent kit.

### Cellular uptake of peptide/Cy5-labeled pDNA complexes

Huh-7 cells were seeded on 24-well culture plates (10000 cells/well) and incubated in 400 μL of DMEM 10% FBS. The medium was then replaced with fresh medium containing 10% FBS, and a peptide/Cy5-labeled pDNA complex solution was added to each well (1 μg pDNA/well). After a 24-h incubation, the medium was removed and cells were washed with ice-cold PBS and then trypsinized. Subsequent operations were same as those in Section *Cellular uptake of peptides*.

### Cell viability with peptide/pDNA complexes

Huh-7 cells were seeded on 96-well culture plates (2500 cells/well) and incubated in 100 μL of DMEM containing 10% FBS. The medium was then replaced with fresh medium containing 10% FBS, and a peptide/Cy5-pDNA complex solution was added to each well (0.25 μg pDNA/well). After a 24-h incubation, Cell counting kit-8 was used according to the manufacturer’s protocol. The results are presented as the mean and standard deviation obtained from 4 samples.

### CLSM observations of peptide/Cy5-labeled pDNA complexes with LysoTracker Green

Huh-7 cells were seeded on 8-well chambered cover glasses (Iwaki) (20000 cells/well) and incubated overnight in 200 μL of DMEM containing 10% FBS. The medium was then replaced with fresh medium containing 10% FBS, and a peptide/Cy5-labeled pDNA complex solution was applied to each well (0.5 μg pDNA/well). After a 24-h incubation, the medium was removed and cells were washed 3 times with ice-cold PBS supplemented with heparin (20 units/mL). Intracellular distribution was observed by CLSM after staining late endosomes/lysosomes with LysoTracker Green and nuclei with Hoechst 33342. CLSM observations were performed using LSM 710 (Carl Zeiss) with a Plan-Apochromat 63×/1.4 objective (Carl Zeiss) at an excitation wavelength of 405 nm (UV laser) for Hoechst 33342, 488 nm (Ar laser) for LysoTracker Green, and 633 nm (He-Ne laser) for Cy5-labeled pDNA. The rate of the colocalization of Cy5-labeled pDNA (Cy5-pDNA) with LysoTracker Green was quantified. The colocalization ratio was quantified as follows:


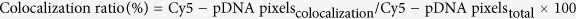


where Cy5-pDNA pixels_colocalization_ represents the number of Cy5-pDNA pixels colocalizing with LysoTracker Green in the cell, and Cy5-pDNA pixels_total_ represents the number of all Cy5-pDNA pixels in the cell. The results are represented as the mean and standard deviation obtained from 20 cells.

## Additional Information

**How to cite this article**: Oba, M. *et al.* A Cell-Penetrating Peptide with a Guanidinylethyl Amine Structure Directed to Gene Delivery. *Sci. Rep.* **6**, 19913; doi: 10.1038/srep19913 (2016).

## Supplementary Material

Supplementary Information
