# Supplementary material for: A Cell-Penetrating Peptide with a Guanidinylethyl Amine Structure Directed to Gene Delivery
Source: Sci Rep. 2016 Jan 27;6:19913. doi: 10.1038/srep19913 (PMC4728608; doi:10.1038/srep19913)
Supplement: Supplementary Information [file srep19913-s1.pdf]

A Cell-Penetrating Peptide with a Guanidinylethyl Amine Structure Directed to Gene Delivery

Makoto Oba *, Takuma Kato, Kaori Furukawa, Masakazu Tanaka

Graduate School of Biomedical Sciences, Nagasaki University, 1-14 Bunkyo-machi, Nagasaki 852-8521, Japan.

*Corresponding author

E-mail: moba@nagasaki-u.ac.jp

Table of contents

General S3

Scheme S1 S4

Scheme S2 S4

Scheme S3 S4

Synthesis of Fmoc-L-Lys[Boc,AEt(Boc)]-OH **12** S5

Synthesis of Fmoc-L-Lys[Boc,GEt$(Boc)_2$]-OH **18** S7

Synthesis of model compound **22** S10

Figure S1 S12

Figure S2 S13

Synthesis and characterization of peptides S14

Figure S3 S15

Table S1 S16

Zeta-potential measurements

Dynamic light scattering (DLS) measurements

NMR spectra S18

Reference S26

General. Optical rotations $[\alpha]^{rt}_D$ were measured with a JASCO DIP-370 polarimeter (JASCO, Tokyo, Japan) using a 0.5 dm cell. Infrared (IR) spectra were recorded on a Shimazu IRAffinity-1 spectrometer (Shimadzu Corporation, Kyoto, Japan) for conventional measurement (neat or KBr). ^{1}H NMR and ^{13}C NMR spectra were determined at JEOL AL 400 (JEOL Ltd, Tokyo, Japan). FAB-MS spectra were taken on a JEOL JMS-700N spectrometer. MALDI-TOF-MS spectra were taken on an Ultraflex (Bruker Daltonics, Fermont, CA). Piperidine, 2-(*tert*-butoxycarbonylamino)-1-ethanol, *N*-ethylethylenediamine, and 1,3-bis(*tert*-butoxycarbonyl)-2-(trifluoromethanesulfonyl)guanidine, were purchased from Tokyo Chemical Industry Co., Ltd. (Tokyo, Japan). Cbz-L-Lys-OMe was obtained from Watanabe Chemical Industries Co., Ltd. (Hiroshima, Japan). Fmoc-Lys(Boc), Fmoc-L-Arg(Pbf), Fmoc-Gly, and CLEAR-Amide resin were purchased from the Peptide Institute., Inc. (Osaka, Japan). COMU was the product of Novabiochem (Tokyo, Japan). CF, TMR, and Dulbecco's modified Eagle's medium (DMEM) were obtained from Sigma-Aldrich Co. (St. Louis, MO). Boc_2O, 5% Pd-C, DIPEA, heparin, and Cell lysis buffer M were purchased from Wako Pure Chem. Co., Inc. (Osaka, Japan). Diethyl ether was the product from Kanto Chemical Co., Inc. (Tokyo, Japan). Fmoc-OSu and TFA were obtained from Nacalai Tesque, Co., Inc. (Kyoto, Japan). Hoechst 33342 was purchased from Dojindo Laboratories (Kumamoto, Japan). LysoTracker Green was obtained from Molecular Probes (Eugene, OR). The micro bicinchoninic acid (BCA) protein assay reagent kit was from Thermo Fisher Scientific, Inc. (Rockford, IL). Plasmid pCAcc+Luc, coding for firefly luciferase under the control of the CAG promoter, was provided by the RIKEN Gene Bank (Tsukuba, Japan), amplified in competent DH5a *Escherichia coli*, and then purified using a HiSpeed Plasmid MaxiKit purchased from Qiagen Sciences (Germantown, MD). The luciferase assay kit was a product of Promega (Madison, WI). pDNA was labeled with Cy5 using the Label IT® Tracker™ Intracellular Nucleic Acid Localization Kit obtained from Mirus Bio Co. (Madison, WI).

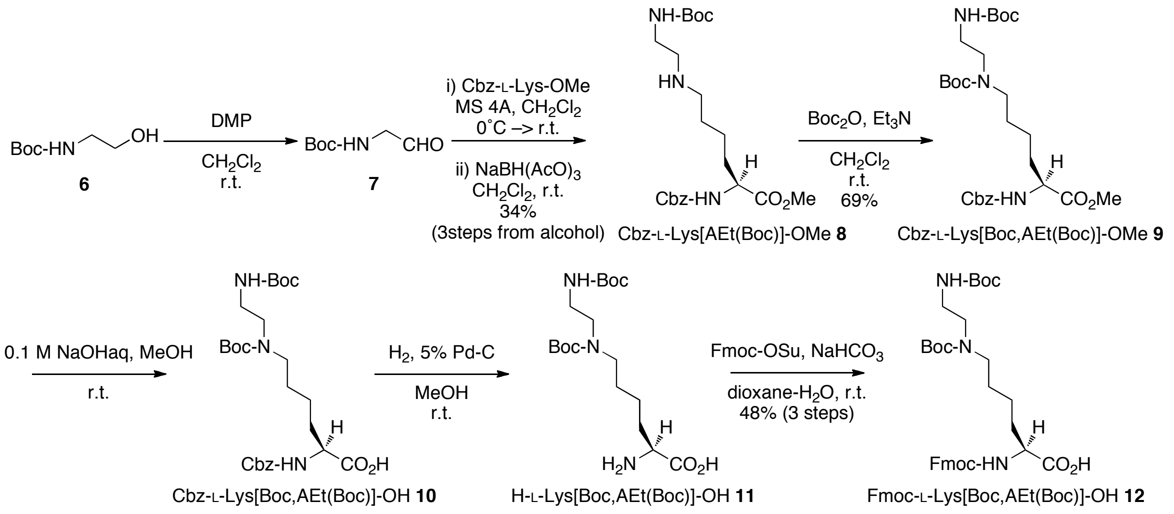

Scheme S1. Synthesis of Fmoc-L-Lys[Boc,AEt(Boc)]-OH **12**

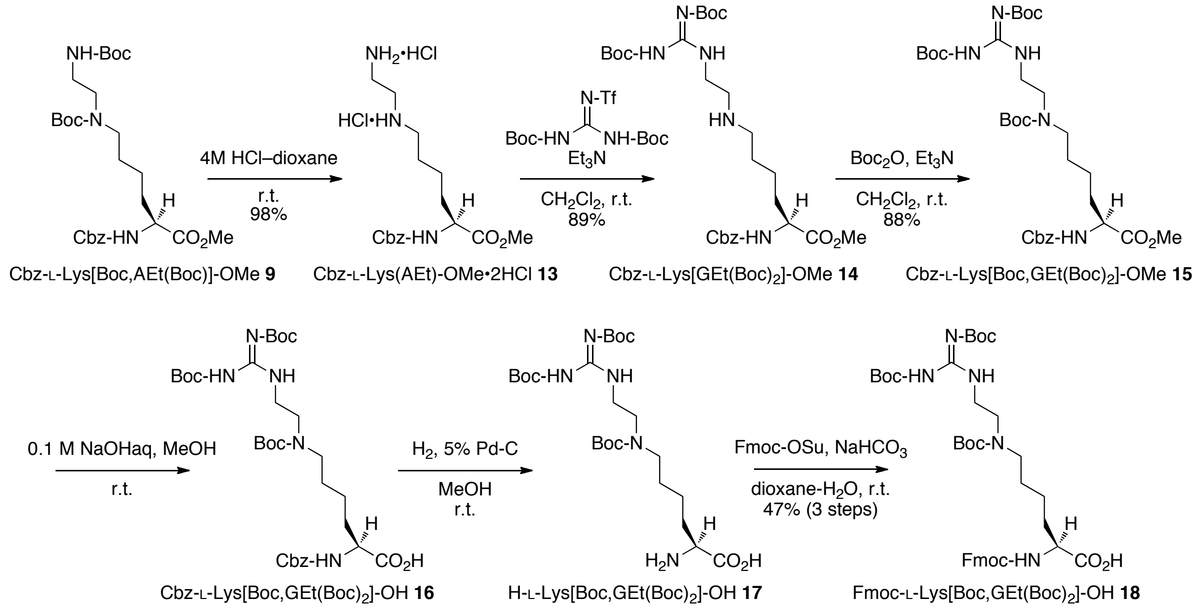

Scheme S2. Synthesis of Fmoc-L-Lys[Boc,GEt(Boc)$_2$]-OH **18**

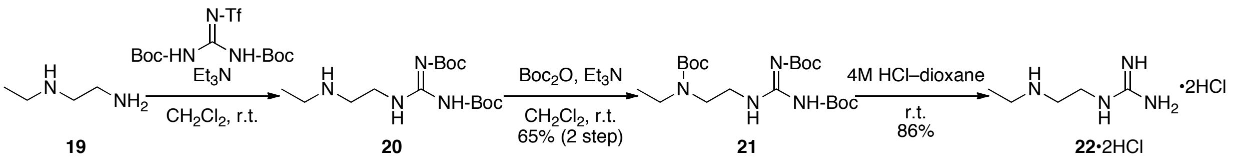

Scheme S3. Synthesis of model compound **22**

Synthesis of Fmoc-L-Lys[Boc,AEt(Boc)]-OH 12 (Scheme S1)

***N*-α-Benzyloxycarbonyl-*N*-ε-*tert*-butoxycarbonyl-2'-aminoethyl-L-lysine methyl ester {Cbz-L-Lys[AEt(Boc)]-OMe, (8)}.** Dess-Martin periodinane (DMP; 9.08 g, 21.4 mmol) was added to a stirred solution of 2-(*tert*-butoxycarbonylamino)-1-ethanol **6** (2.88 g, 17.8 mmol) in CH_2Cl_2 (100 mL), and the solution was stirred at room temperature for 2 h. Saturated aqueous $NaHCO_3$-$Na_2S_2O_3$ solution (100 mL) was added and then stirred at room temperature for 30 min. The solution was extracted with $CHCl_3$, dried over $MgSO_4$, and evaporated in vacuo to leave a crude aldehyde. A solution of crude aldehyde, Cbz-L-Lys-OMe (5.25 g, 17.8 mmol), and MS4Å (1 g) in CH_2Cl_2 (100 mL) was stirred at 0˚C for 1 h. Sodium triacetoxyborohydride (4.80 g, 22.7 mmol) was added to a stirred solution and then stirred at room temperature overnight. The solution was extracted with $CHCl_3$ and dried over $MgSO_4$. Removal of the solvent afforded a residue, which was purified by column chromatography on silica gel (10% MeOH in $CHCl_3$) to give Cbz-L-Lys[AEt(Boc)]-OMe **8** (2.63 g, 34%) as a colorless oil: $[\alpha]_D^{25}$ = +2.92 (*c* 1.35, $CHCl_3$); IR (neat) ν 3345, 2955, 1715, 1697, 1520, 1454, 1400, 1254, 1215, 1169, 1049 cm^{-1}; ^1H NMR (400 MHz, $CDCl_3$) δ 7.29–7.36 (m, 5H), 5.75 (br s, 1H), 5.69 (br s, 1H), 5.10 (s, 2H), 4.35 (m, 1H), 3.74 (s, 3H), 3.44 (br s, 1H), 3.31–3.35 (m, 2H), 2.84–2.90 (m, 2H), 2.74 (t, J = 7.10 Hz, 2H), 1.96–1.98 (m, 2H), 1.83 (m, 1H), 1.60–1.71 (m, 3H), 1.43 (s, 9H); ^{13}C NMR (100 MHz, $CDCl_3$) δ 172.7, 156.5, 156.1, 136.2, 128.5, 128.13, 128.09, 80.0, 67.0, 53.6, 52.6, 48.5, 47.8, 37.3, 31.7, 28.4, 25.5, 22.4; FAB(+)HRMS calcd for $C_{22}H_{36}N_3O_6$ $[M^+ + H]$: 438.2604; found: 438.2596.

***N*-α-Benzyloxycarbonyl-*N*-ε-(*tert*-butoxycarbonyl-2'-aminoethyl)-*N*-ε-*tert*-butoxycarbonyl-L-lysine methyl ester {Cbz-L-Lys[Boc,AEt(Boc)]-OMe, (9)}.** Boc_2O (583 mg, 2.67 mmol) and Et_3N (270 mg, 2.67 mmol) were added to a stirred solution of Cbz-L-Lys[AEt(Boc)]-OMe **8** (973 mg, 2.22 mmol) in CH_2Cl_2 (30 mL), and the solution was stirred at room temperature for 5 h.

Removal of the solvent afforded a residue, which was purified by column chromatography on silica gel (40% EtOAc in *n*-hexane) to give Cbz-L-Lys[Boc,AEt(Boc)]-OMe **9** (820 mg, 69%) as a colorless oil: $[\alpha]_D^{25}$ = +1.99 (*c* 2.06, $CHCl_3$); IR (neat) *v* 3345, 2974, 1725, 1710, 1686, 1520, 1416, 1366, 1250, 1169, 1065 cm^{-1}; ^1H NMR (400 MHz, $CDCl_3$) *δ* 7.28–7.36 (m, 5H), 5.37–5.52 (m, 1H), 5.10 (s, 2H), 4.80–5.04 (m, 1H), 4.35 (m, 1H), 3.74 (s, 3H), 3.17–3.24 (m, 6H), 1.84 (m, 1H), 1.69 (m, 1H), 1.50–1.53 (m, 2H), 1.44 (s, 9H), 1.43 (s, 9H), 1.24–1.36 (m, 2H); ^{13}C NMR (100 MHz, $CDCl_3$) *δ* 172.8, 156.0, 155.8, 136.2, 128.3, 79.7, 69.1, 66.8, 53.7, 52.2, 47.3, 46.3, 39.4, 32.2, 31.7, 28.2, 27.4, 22.3; FAB(+)HRMS calcd for $C_{27}H_{44}N_3O_8$ [M^+ + H]: 538.3128; found: 538.3132.

***N*-α-(9-Fluorenylmethoxycarbonyl)-*N*-ε-(*tert*-butoxycarbonyl-2'-aminoethyl)-*N*-ε-*tert*-butoxy carbonyl-L-lysine {Fmoc-L-Lys[Boc,AEt(Boc)]-OH, (12)}.** A solution of 0.1 M aqueous NaOH (27.1 mL, 2.71 mmol) was added to a stirred solution of Cbz-L-Lys[Boc,AEt(Boc)]-OMe **9** (1.22 g, 2.26 mmol) in MeOH (10 mL), and the solution was stirred at room temperature for 2 h. After removal of MeOH, the solution was acidfied with 1 M aqueous $NaHSO_4$ to pH 2–3, extracted with EtOAc, dried over Na_2SO_4, and evaporated in vacuo to leave a crude carboxylic acid **10** (1.00 g, 84%). A mixture of the crude carboxylic acid **10** (1.00 g, 1.91 mmol) and 5% Pd-C (300 mg) in MeOH (30 mL) was vigorously stirred under H_2 atmosphere at room temperature. After being stirred overnight, the Pd-C catalyst was filtered off, and the filtrate was evaporated in vacuo to leave a crude amino acid **11** (719 mg, 97%). A solution of Fomc-OSu (685 mg, 2.03 mmol) in dioxane (10 mL) was added to a stirred solution of the crude amino acid **11** (719 mg, 1.85 mmol) and $NaHCO_3$ (465 mg, 5.54 mmol) in water (30 mL), and the solution was stirred at room temperature overnight. After removal of the dioxane, the solution was acidfied with citric acid, extracted with EtOAc, and dried over Na_2SO_4. Removal of the solvent afforded a white solid, which was purified column chromatography on silica gel. The fraction eluted with 10% MeOH in

$CHCl_3$ gave Fmoc-L-Lys[Boc,AEt(Boc)]-OH **12** (670 mg, 48%) as colorless crystals: M.p. 67–69°C; $[\alpha]_D^{26}$ = +13.9 (*c* 0.86, $CHCl_3$); IR (KBr) *v* 3329, 3233, 2936, 2766, 2731, 2704, 2442, 1740, 1690, 1543, 1504, 1454, 1273, 1169, 1038, 999 cm^{-1}; ^1H NMR (400 MHz, $CDCl_3$) δ 8.57 (s, 1H), 7.74 (d, *J* = 7.6 Hz, 2H), 7.53–7.70 (m, 2H), 7.37 (t, *J* = 7.4 Hz, 2H), 7.28 (t, *J* = 7.4 Hz, 2H), 5.72–6.13 (m, 1H), 4.92–5.23 (m, 1H), 4.35–4.46 (m, 3H), 4.20 (t, *J* = 6.8 Hz, 1H), 3.15–3.26 (m, 3H), 1.25–1.91 (m, 6H), 1.44 (s, 9H), 1.42 (s, 9H); ^{13}C NMR (100 MHz, $CDCl_3$) δ 175.2, 156.4, 156.1, 143.8, 143.7, 141.2, 127.6, 127.0, 125.1, 119.9, 80.1, 79.5, 67.0, 53.6, 47.0, 46.2, 39.3, 31.6, 28.3, 27.5, 22.2; FAB(+)HRMS calcd for $C_{33}H_{46}N_3O_8$ [M^+ + H]: 612.3285; found: 612.3292.

Synthesis of Fmoc-L-Lys[Boc,GEt(Boc)$_2$]-OH 18 (Scheme S2)

***N*-α-Benzyloxycarbonyl-*N*-ε-2'-aminoethyl-L-lysine methyl ester dihydrochloride {Cbz-L-Lys(AEt)}-OMe•2HCl, (13)}.** A solution of Cbz-L-Lys[Boc,AEt(Boc)]-OMe **9** (387 mg, 0.720 mmol) in 4 M HCl/dioxane (7.2 mL) was stirred at room temperature for 2 h. Removal of the solvent afforded Cbz-L-Lys(AEt)-OMe•2HCl **13** (289 mg, 98%) as colorless crystals: M.p. 149–151 °C; $[\alpha]_D^{27}$ = –14.3 (*c* 1.11, MeOH); IR (KBr) *v* 3341, 2978, 2936, 1720, 1524, 1450, 1420, 1366, 1250, 1165, 1076 cm^{-1}; ^1H NMR (400 MHz, CD_3OD) δ 7.22–7.29 (m, 5H), 5.02 (s, 2H), 4.13 (m, 1H), 3.65 (s, 3H), 3.23–3.33 (m, 4H), 3.01 (t, *J* = 7.4 Hz, 2H), 1.62–1.85 (m, 4H), 1.37–1.50 (m, 2H); ^{13}C NMR (100 MHz, CD_3OD) δ 174.3, 158.6, 138.1, 129.5, 129.0, 128.8, 67.7, 55.2, 52.8, 49.6, 45.7, 36.9, 31.9, 26.7, 23.8; FAB(+)HRMS calcd for $C_{17}H_{28}N_3O_4$ [M^+ + H]: 338.2080; found: 338.2032.

***N*-α-Benzyloxycarbonyl-*N*-ε-[*N'*,*N''*-bis(*tert*-butoxycarbonyl)-2'-guanidinyl]ethyl-L-lysine methyl ester {Cbz-L-Lys[GEt(Boc)$_2$]-OMe, (14)}.** Cbz-L-Lys(AEt)-OMe•2HCl **13** (289 mg, 0.681 mmol) was added to a stirred solution of

1,3-bis(*tert*-butoxycarbonyl)-2-(trifluoromethanesulfonyl)guanidine (320 mg, 0.817 mmol) and Et_3N (83 mg, 0.817 mmol) in CH_2Cl_2 (10 mL), and the solution was stirred at room temperature overnight. Removal solvent afforded a residue, which was readily purified by short column chromatography on silica gel (8% MeOH in $CHCl_3$) to give Cbz-L-Lys[GEt(Boc)$_2$]-OMe **14** (362 mg, 89%) as a colorless oil: ^{1}H NMR (400 MHz, $CHCl_3$) δ 11.42 (br s, 1H), 8.76 (br s, 1H), 7.29–7.36 (m, 5H), 5.54 (br s, 1H), 5.53 (d, $J = 7.6$ Hz, 1H), 5.11 (s, 2H), 4.35 (m, 1H), 3.74 (s, 3H), 3.53–3.60 (m, 2H), 2.98–3.04 (m, 2H), 2.75–2.79 (m, 2H), 1.85 (m, 1H), 1.66–1.75 (m, 3H), 1.50 (s, 9H), 1.48 (s, 9H), 1.21–1.58 (m, 2H).

***N*-α-Benzyloxycarbonyl-*N*-ε-[*N'*,*N''*-bis-(*tert*-butoxycarbonyl)-2'-guanidinyl]ethyl-*N*-ε-*tert*-butoxycarbonyl-L-lysine methyl ester {Cbz-L-Lys[Boc,GEt(Boc)$_2$]-OMe, (15)}.** A mixture of Cbz-L-Lys[GEt(Boc)]$_2$-OMe **14** (1.88 g, 3.24 mmol), Boc_2O (849 mg, 3.89 mmol), and Et_3N (394 mg, 3.89 mmol) in CH_2Cl_2 (30 mL) was stirred at room temperature for 4 h. Removal of solvent afforded a residue, which was purified by column chromatography on silica gel (40% EtOAc in *n*-hexane) to give Cbz-L-Lys[Boc,GEt(Boc)]$_2$-OMe **15** (1.94 g, 88%) as a colorless oil: $[\alpha]_D^{21}$ = +4.59 (*c* 1.14, $CHCl_3$); IR (neat) *v* 3333, 2978, 2936, 1728, 1690, 1682, 1630, 1616, 1574, 1535, 1415, 1366, 1335, 1254, 1231, 1157, 1134, 1096, 1060, 1022 cm^{-1}; ^{1}H NMR (400 MHz, $CHCl_3$) δ 11.48 (br s, 1H), 8.44 (m, 1H), 7.31–7.36 (m, 5H), 5.36–5.52 (m, 1H), 5.10 (s, 2H), 4.34 (m, 1H), 3.73 (s, 3H), 3.50–3.56 (m, 2H), 3.34–3.42 (m, 2H), 3.12–3.24 (m, 1H), 1.84 (m, 1H), 1.70 (m, 1H), 1.49 (s, 9H), 1.48 (s, 9H), 1.44 (s, 9H), 1.27–1.40 (m, 4H); ^{13}C NMR (100 MHz, $CHCl_3$) δ 172.8, 163.4, 156.3, 155.9, 155.6, 153.0, 136.2, 128.4, 128.0, 83.0, 79.9, 79.1, 66.8, 53.7, 52.2, 46.0, 45.6, 39.4, 31.8, 28.23, 28.18, 28.1, 27.9, 27.3, 22.2; FAB(+)HRMS calcd for $C_{33}H_{54}N_5O_{10}$ [M^+ + H]: 680.3871; found: 680.3863.

***N*-α-(9-Fluorenylmethoxycarbonyl)-*N*-ε-[*N'*,*N''*-bis-(*tert*-butoxycarbonyl)-2'-guanidinyl] ethyl-*N*-ε-*tert*-butoxycarbonyl-L-lysine {Fmoc-L-Lys[Boc,GEt(Boc)$_2$]-OH, (18)}.** A solution of 0.1 M aqueous NaOH (30.4 mL, 3.04 mmol) was added to a stirred solution of Cbz-L-Lys[Boc,GEt(Boc)$_2$]-OMe **15** (1.88 g, 2.76 mmol) in MeOH (30 mL), and the solution was stirred at room temperature for 24 h. After removal of MeOH, the solution was acidfied with citric acid to pH 2–3, extracted with EtOAc, dried over Na_2SO_4, and evaporated in vacuo to leave a crude carboxylic acid **16** (1.66 g, 90%). A mixture of the crude carboxylic acid **16** (1.66 g, 2.49 mmol) and 5% Pd-C (500 mg) in MeOH (30 mL) was vigorously stirred under H_2 atmosphere at room temperature. After being stirred overnight, the Pd-C catalyst was filtered off, and the filtrate was evaporated in vacuo to leave a crude amino acid **17** (1.33 g, quantitatively). A solution of Fomc-OSu (925 mg, 2.74 mmol) in dioxane (20 mL) was added to a stirred solution of the crude amino acid **17** (1.33 g, 2.49 mmol) and $NaHCO_3$ (628 mg, 7.48 mmol) in water (20 mL), and the solution was stirred at room temperature overnight. After removal of the dioxane, the solution was acidfied with citric acid, extracted with EtOAc, and dried over Na_2SO_4. Removal of the solvent afforded a white solid, which was purified column chromatography on silica gel. The fraction eluted with 3% MeOH in $CHCl_3$ gave Fmoc-L-Lys[Boc,GEt(Boc)$_2$]-OH **18** (972 mg, 52%) as colorless crystals: M.p. 93–94°C; $[\alpha]_D^{20}$ = +12.1 (*c* 1.41, $CHCl_3$); IR (KBr) *ν* 3333, 2979, 2936, 1721, 1686, 1639, 1620, 1420, 1366, 1331 cm^{-1}; ^{1}H NMR (400 MHz, $CDCl_3$) *δ* 11.53 (br s, 1H), 8.49 (m, 1H), 7.76 (d, *J* = 7.2 Hz, 2H), 7.60 (d, *J* = 6.0 Hz, 2H), 7.39 (t, *J* = 7.2 Hz, 2H), 7.31 (t, *J* = 7.2 Hz, 2H), 6.23 (br s, 1H), 5.61 (m, 1H), 4.35–4.50 (m, 3H), 4.22 (t, *J* = 6.8 Hz, 1H), 3.16–3.51 (m, 6H), 1.89–1.97 (m, 2H), 1.48 (s, 9H), 1.47 (s, 9H), 1.45 (s, 9H), 1.20–1.60 (m, 4H); ^{13}C NMR (100 MHz, $CDCl_3$) *δ* 174.8, 163.1, 156.5, 156.0, 155.7, 152.8, 143.9, 143.8, 141.3, 127.7, 127.0, 119.9, 83.3, 80.4, 79.6, 67.0, 53.5, 47.1, 46.5, 46.0, 39.6, 31.1, 28.3, 28.2, 27.3, 21.7; FAB(+)HRMS calcd for $C_{39}H_{56}N_5O_{10}$ [M^+ + H]: 754.4027; found: 754.4050.

Synthesis of model compound 22 (Scheme S3)

1-[*N'*,*N''*-bis-(*tert*-butoxycarbonyl)-guanidinyl]-2-[N'''-(*tert*-butoxycarbonyl)-N'''-ethylamino] ethane (21). *N*-Ethylethylenediamine **19** (205 mg, 2.32 mmol) was added to a stirred solution of 1,3-bis(*tert*-butoxycarbonyl)-2-(trifluoromethanesulfonyl)guanidine (1000 mg, 2.56 mmol) and Et_3N (259 mg, 2.32 mmol) in CH_2Cl_2 (20 mL), and the solution was stirred at room temperature overnight [S1]. Removal solvent afforded a residue, which was readily purified by short column chromatography on silica gel (8% MeOH in $CHCl_3$) to give compound **20** (768 mg, quantitatively). Boc_2O (760 mg, 3.49 mmol) and Et_3N (353 mg, 3.49 mmol) were added to a stirred solution of compound **20** (768 mg, 2.32 mmol) in CH_2Cl_2 (30 mL), and the solution was stirred at room temperature for 2 h. Removal of the solvent afforded a residue, which was purified by column chromatography on silica gel (10% EtOAcin *n*-hexane) to give compound **21** (654 mg, 65%) as colorless crystals: M.p 102–103 ˚C; IR (KBr) ν 3321, 3136, 2978, 2932, 1813, 1740, 1686, 1628, 1562, 1477, 1420, 1362, 1319, 1288, 1254, 1157 cm^{-1}; ^1H NMR (400 MHz, $CDCl_3$) δ 11.32 (br s, 1H), 8.23–8.29 (m, 1H), 3.33–3.36 (m, 2H), 3.19–3.23 (m, 2H), 3.00–3.10 (m, 2H), 1.32 (s, 9H), 1.28 (s, 9H), 1.25 (s, 9H); ^{13}C NMR (100 MHz, $CDCl_3$) δ 163.5, 156.3, 152.9, 82.9, 79.7, 79.1, 45.2, 41.8, 39.6, 28.2, 28.0, 27.4, 13.4; FAB(+)HRMS calcd for $C_{20}H_{39}N_4O_6$ [M^+ + H]: 431.2870; found: 431.2870.

1-Ethylamino-2-guanidinylethane hydrochloride (22•2HCl). A solution of compound **21** (568 mg, 1.72 mmol) in 4 M HCl/dioxane (5 mL) was stirred at room temperature overnight. Removal of the solvent afforded compound **22**•2HCl (299 mg, 86%) as colorless crystals: M.p. 67–68 ˚C; IR (KBr) ν 3470, 3150, 2980, 2810, 2460, 2360, 2190, 2060, 1650, 1440 cm^{-1}; ^1H NMR (400 MHz, $CDCl_3$) δ 3.56 (t, J = 6.2 Hz, 2H), 3.19 (t, J = 6.1 Hz, 2H), 3.08 (q, J = 7.3 Hz, 2H), 1.31 (t, J = 7.3

Hz, 3H); ^{13}C NMR (100 MHz, $CDCl_3$) δ 158.9, 47.0, 44.5, 39.1, 11.5; FAB(+)HRMS calcd for $C_5H_{15}N_4$ [M^+ + H]: 131.1275; found: 131.1300.

(a) TMR-Gly-(L-Lys)$_9$-NH_2 **1**

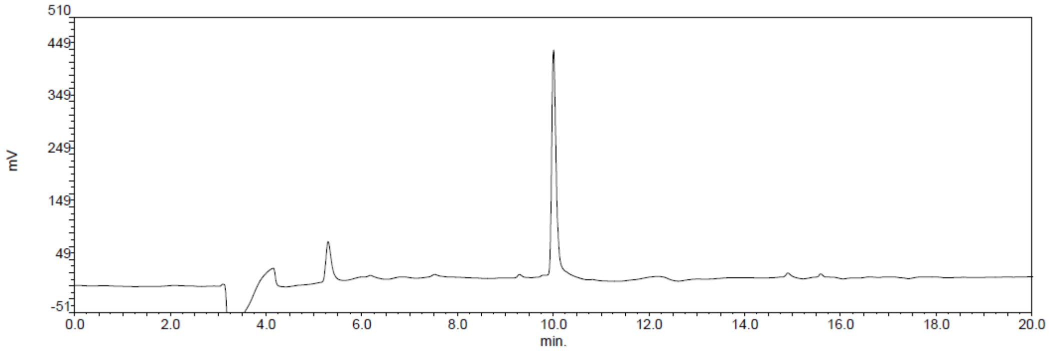

(b) TMR-Gly-(L-Arg)$_9$-NH_2 **2**

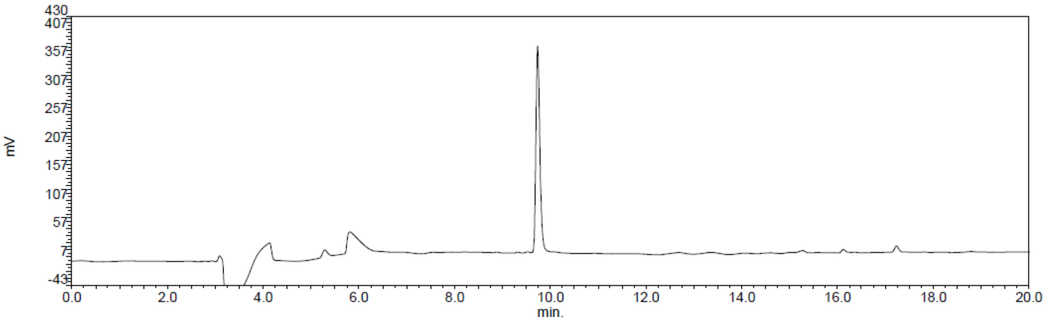

(c) TMR-Gly-[L-Lys(AEt)]$_9$-NH_2 **3**

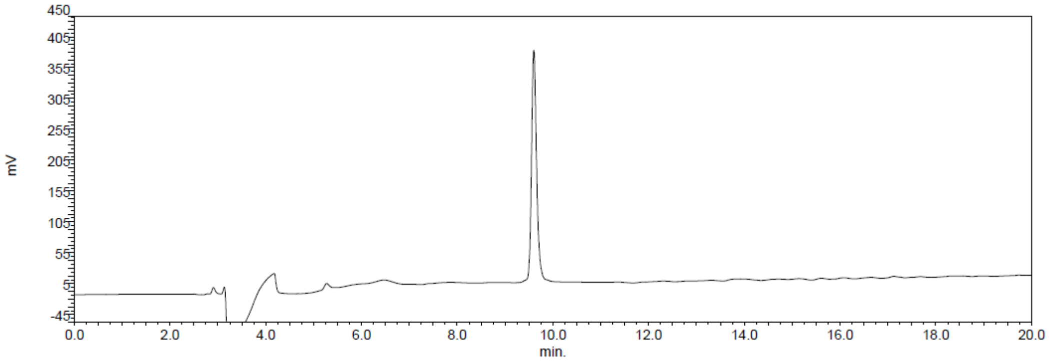

(d) TMR-Gly-[L-Lys(GEt)]$_9$-NH_2 **4**

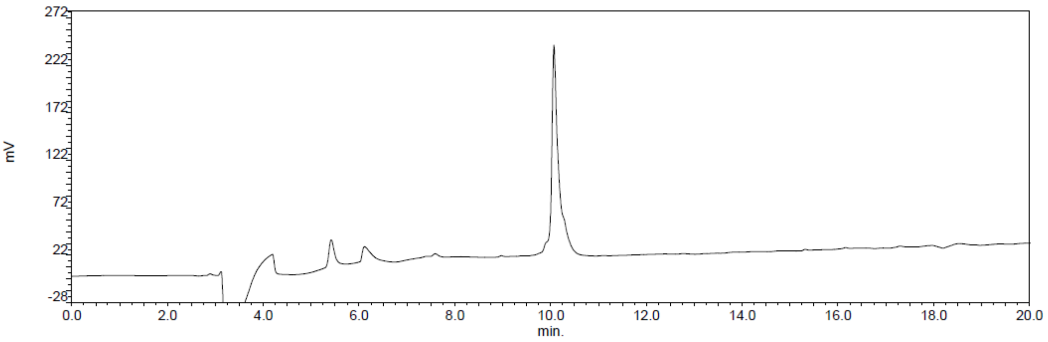

Figure S1. HPLC charts of peptides TMR-Gly-(L-Lys)$_9$-NH_2 **1** (a), TMR-Gly-(L-Arg)$_9$-NH_2 **2** (b), TMR-Gly-[L-Lys(AEt)]$_9$-NH_2 **3** (c), and TMR-Gly-[L-Lys(GEt)]$_9$-NH_2 **4** (d).

(a) TMR-Gly-(L-Lys)$_9$-NH$_2$ **1** (b) TMR-Gly-(L-Arg)$_9$-NH$_2$ **2**

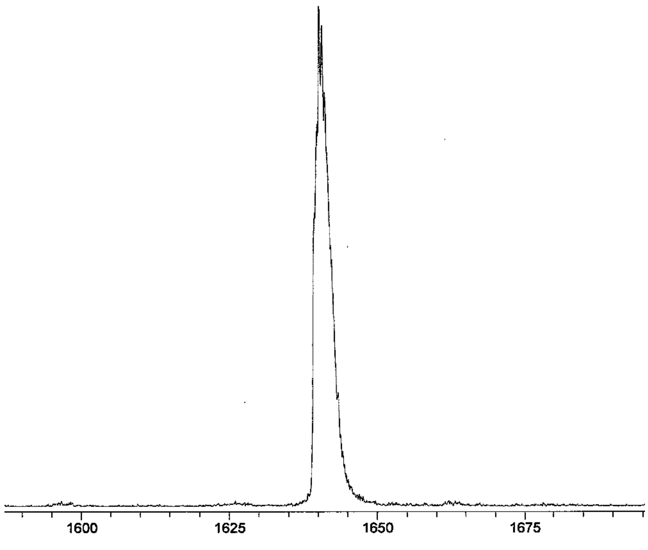

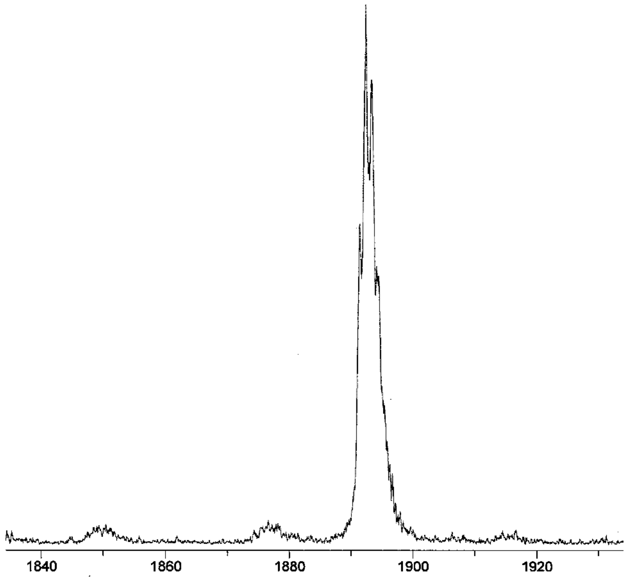

(c) TMR-Gly-[L-Lys(AEt)]$_9$-NH$_2$ **3** (d) TMR-Gly-[L-Lys(GEt)]$_9$-NH$_2$ **4**

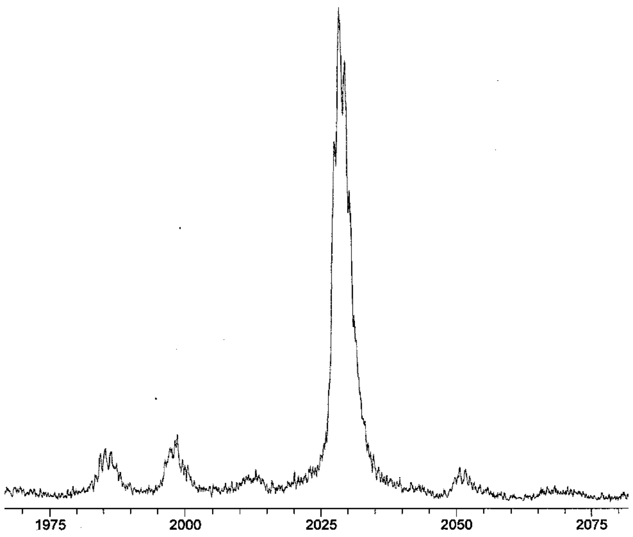

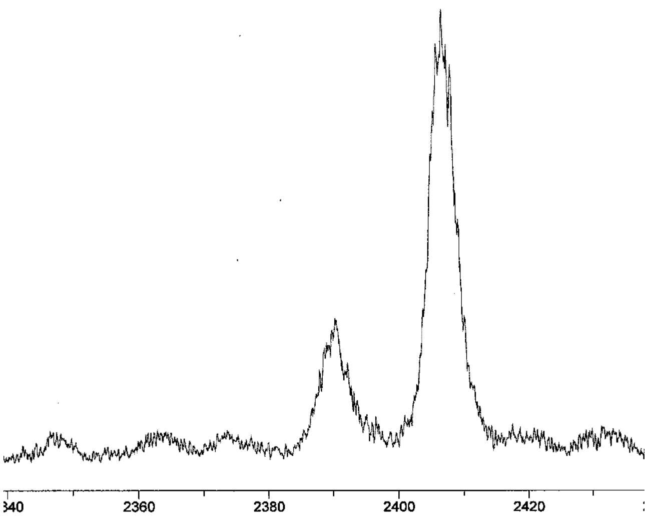

Figure S2. MALDI-TOF-MS charts of peptides TMR-Gly-(L-Lys)$_9$-NH$_2$ **1** (a), TMR-Gly-(L-Arg)$_9$-NH$_2$ **2** (b), TMR-Gly-[L-Lys(AEt)]$_9$-NH$_2$ **3** (c), and TMR-Gly-[L-Lys(GEt)]$_9$-NH$_2$ **4** (d).

Synthesis and characterization of peptides. The peptides were synthesized on solid support by Fmoc solid-phase methods using standard commercially available Rink amide resin and Fmoc-amino acids [S2,S3]. The following describes a representative coupling and deprotection cycle at 10 μmol scales. First, 26.3 mg of CLEAR-Amide resin (loading: 0.38 mmol/g) was soaked overnight in DMF. After DMF had been removed, 20% piperidine in DMF was added to the resin for deprotection. After removing and washing out piperidine, Fmoc-amino acid {Fomc-Gly-OH, Fmoc-L-Lys(Boc)-OH, Fmoc-L-Arg(Pbf)-OH, Fmoc-L-Lys[Boc,AEt(Boc)]-OH **12**, or Fmoc-L-Lys[Boc,GEt(Boc)$_2$]-OH **18**} or 5(6)-tetramethylrhodamine carboxylic acid (3 equiv), COMU (3 equiv), and DIPEA (6 equiv) dissolved in DMF (1.0 mL) were added for the coupling reaction. The resin was then suspended in cleavage cocktail (TFA: 1.9 mL; H_2O: 50 μL; TIS: 50 μL). The TFA solution was evaporated to a small volume and added to cold diethyl ether to precipitate the peptides. The dried crude peptides were dissolved in acetonitrile and/or H_2O, and then purified by RP-HPLC using a COSMOSIL Packed Column 5C_{18}-AR-II (20 ID x 250 mm) (Nacalai). Freeze-drying afforded red crystals, which were characterized by analytical RP-HPLC (COSMOSIL Packed Column 5C_{18}-AR-II, 4.6 ID x 250 mm) and MALDI-TOF-MS (Bruker Daltonics Ultraflex, Fremont, CA). RP-HPLC was performed utilizing JASCO-PU-2089 Plus (JASCO) with a JASCO-2075-Plus as a detector. Solvent A: 0.05% TFA in H_2O; solvent B: 0.05% TFA in acetonitrile. The purification procedure required gradient conditions (from 95% to 50% solvent A over 20 min) with a flow rate of 10 mL/min and detection at 220 nm. The purity of the final compounds was further confirmed using similar RP-HPLC conditions (from 95% to 35% solvent A over 20 min, then from 35% to 10% solvent A over 5 min) with a flow rate of 1.0 mL/min.

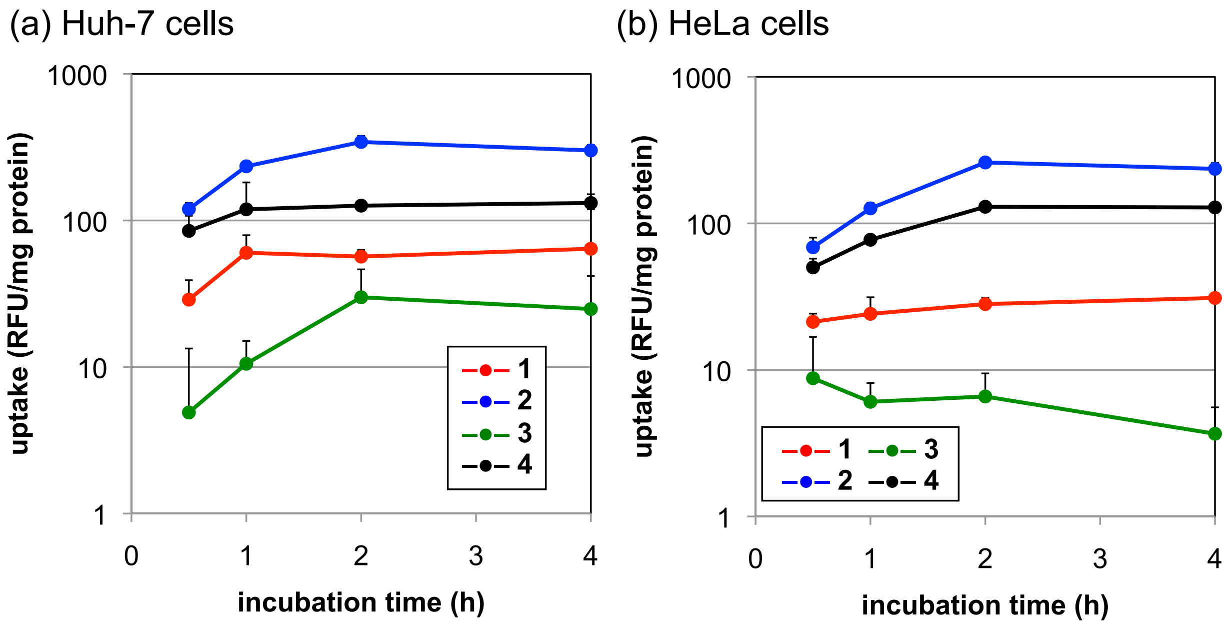

Figure S3. Cellular uptake of peptides **1**–**4** into Huh-7 cells (a) and HeLa cells (b). The incubation time-dependency with a peptide concentration of 1 μM. Error bars represent the standard deviation, n = 3.

Table S1. Zeta-potential and size of each peptide/pDNA complex prepared at various N/P ratios.

	N/P ratio	zeta-potential (mV)	size (nm)	PDI (μ/Γ^2)
Lys-peptide **1**	2	+17.4±0.81	88.8±0.8	0.078±0.011
	4	+19.0±0.79	82.4±0.2	0.086±0.007
	8	+21.0±0.29	77.0±0.7	0.127±0.020
Arg-peptide **2**	2	+16.5±0.38	130±4.0	0.073±0.021
	4	+15.5±0.45	101±0.7	0.072±0.012
	8	+14.8±0.81	91.1±0.7	0.123±0.003
Lys(AEt)-peptide **3**	2	−36.0±0.55	114±0.9	0.113±0.014
	4	+6.40±0.40	231±8.4	0.066±0.029
	8	+6.44±0.43	121±2.1	0.078±0.010
Lys-(GEt)-peptide **4**	2	+18.6±0.53	103±2.7	0.078±0.011
	4	+23.6±0.49	76.6±0.4	0.127±0.016
	8	+25.1±1.15	78.6±0.4	0.154±0.014

Zeta-potential measurements. The zeta-potentials of peptide/pDNA complexes were evaluated by the laser-Doppler electrophoresis method using Nano ZS (ZEN3600, Malvern Instruments, Ltd., UK) with a He-Ne ion laser (633 nm). Zeta-potential measurements were carried out at 25˚C. A scattering angle of 173˚ was used in these measurements. Results were presented as the mean and standard deviation obtained from 3 measurements.

Dynamic light scattering (DLS) measurements. The sizes of the peptide/pDNA complexes were evaluated by DLS using Nano ZS. A He-Ne ion laser (633 nm) was used as the incident beam. Light scattering data were obtained at a detection angle of 173˚ and temperature of 25˚C

and were subsequently analyzed by the cumulant method to obtain the hydrodynamic diameters and polydispersity indices (PDI) (μ/Γ^2) of the complexes. Results were presented as the mean and standard deviation obtained from 3 measurements.

^1H NMR spectrum of **8**

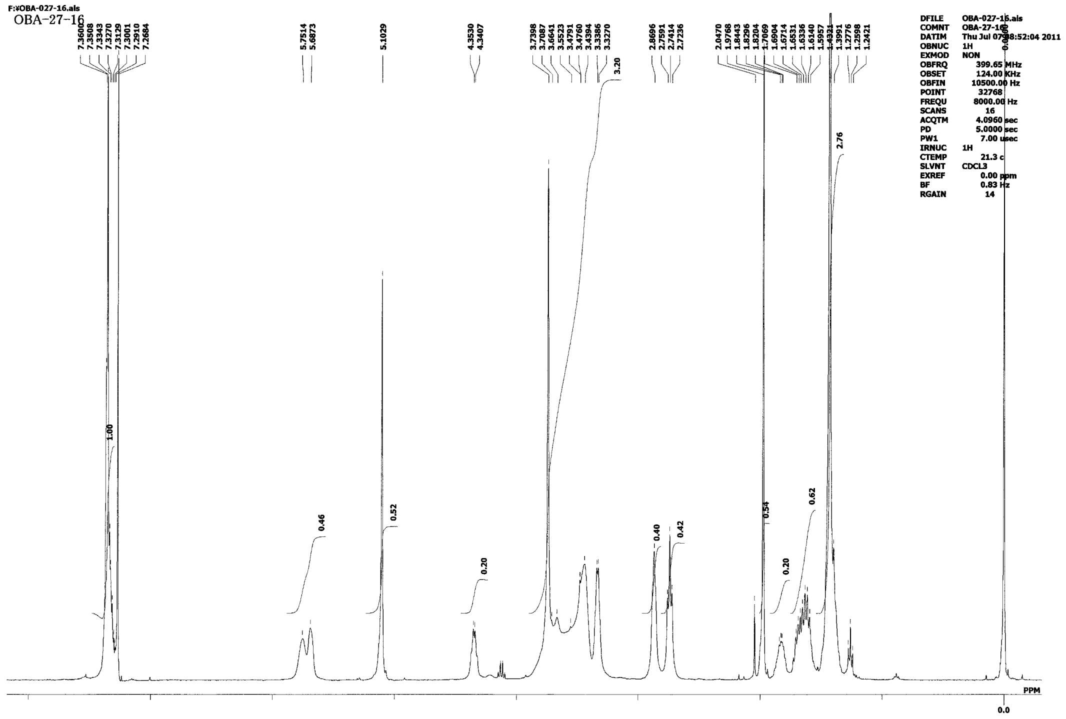

^{13}C NMR spectrum of **8**

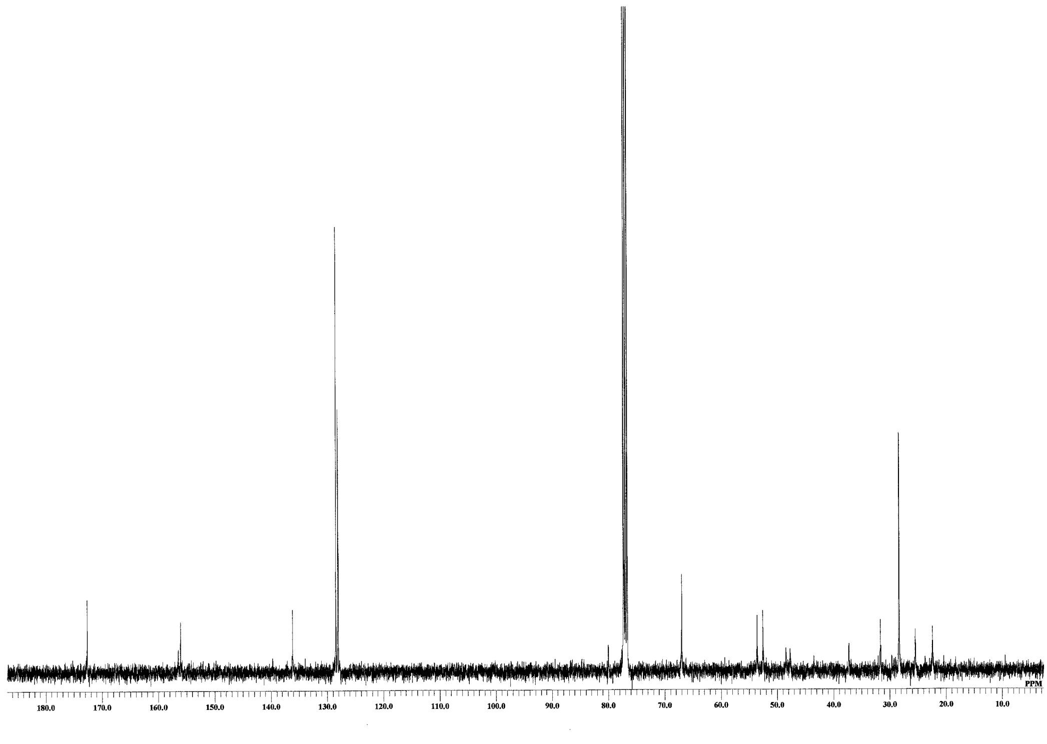

^{1}H NMR spectrum of **9**

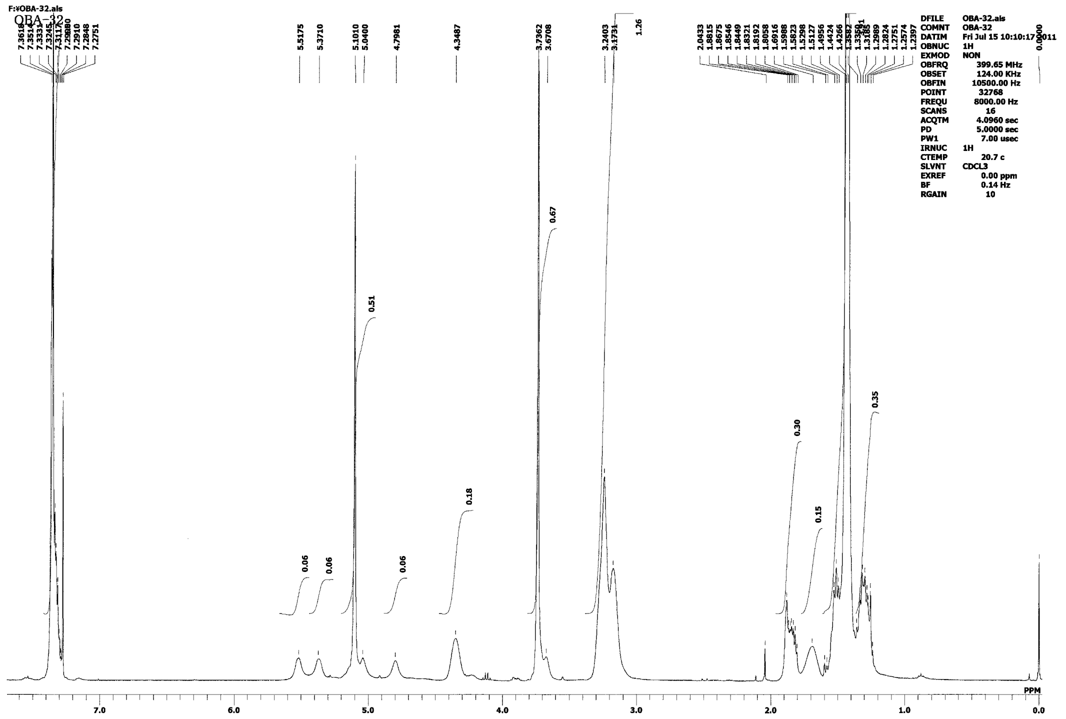

^{13}C NMR spectrum of **9**

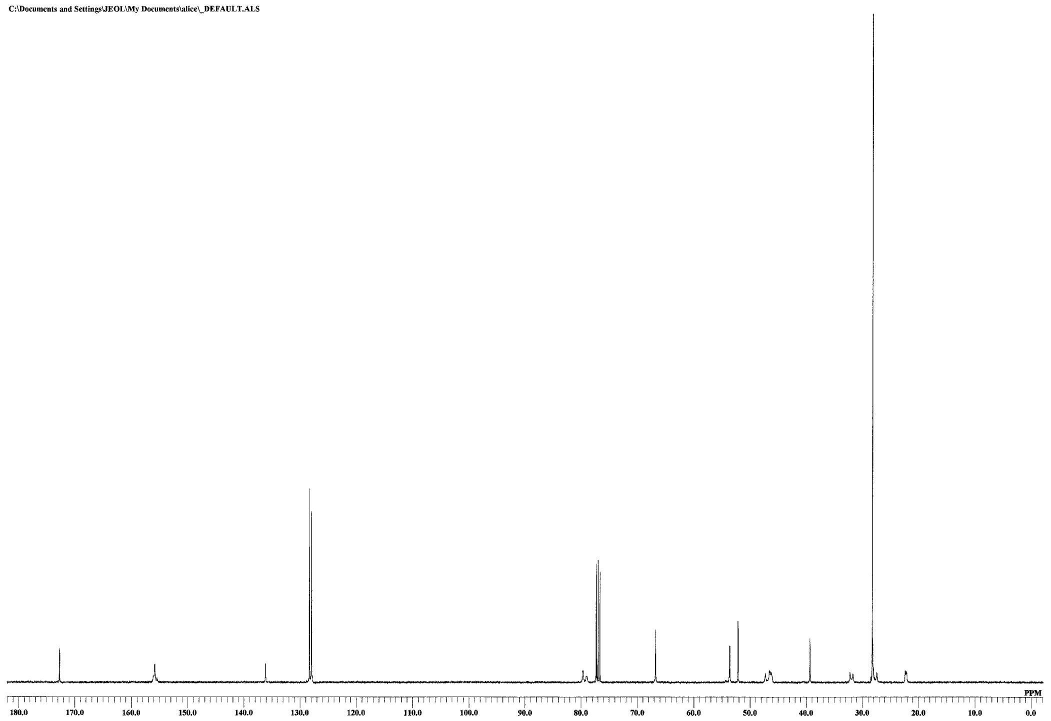

^{1}H NMR spectrum of **12**

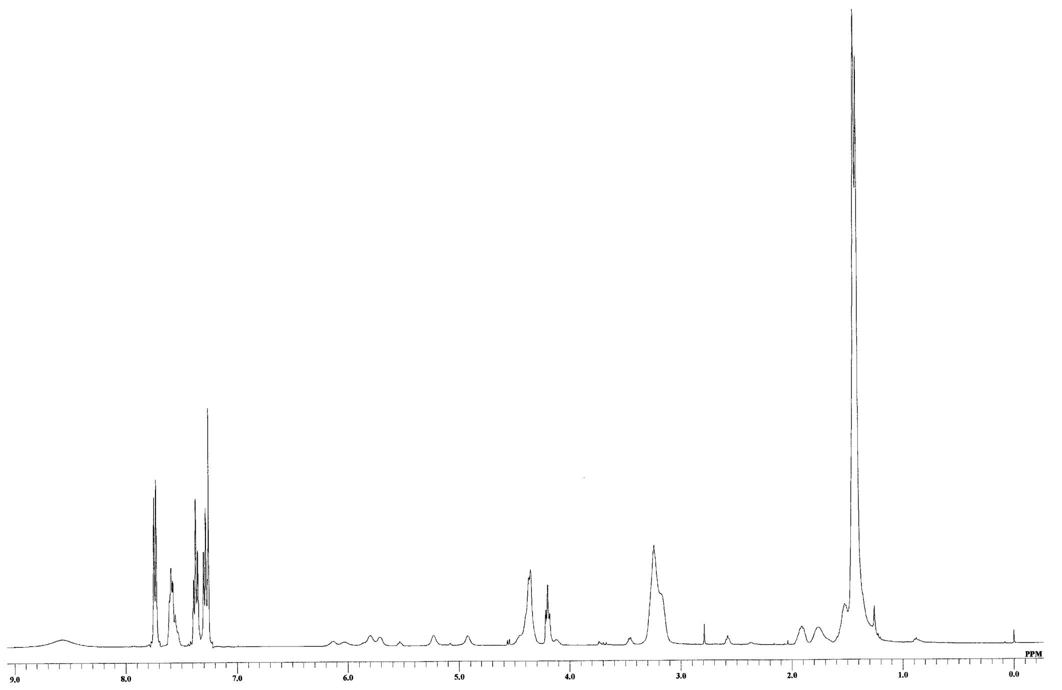

^{13}C NMR spectrum of **12**

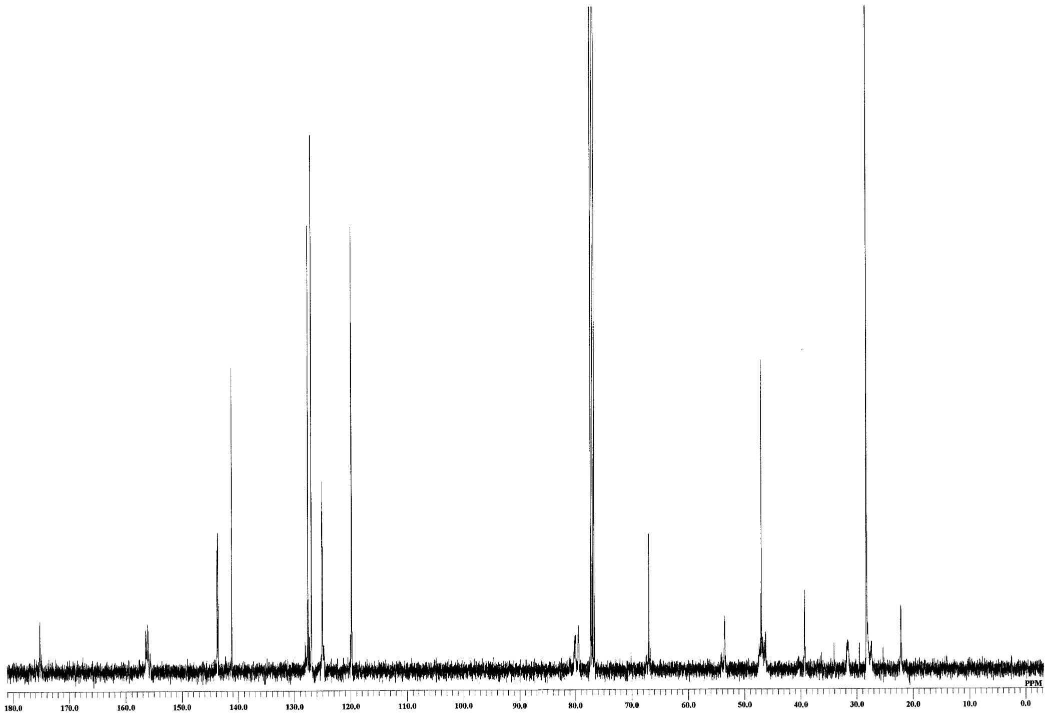

^{1}H NMR spectrum of **13**

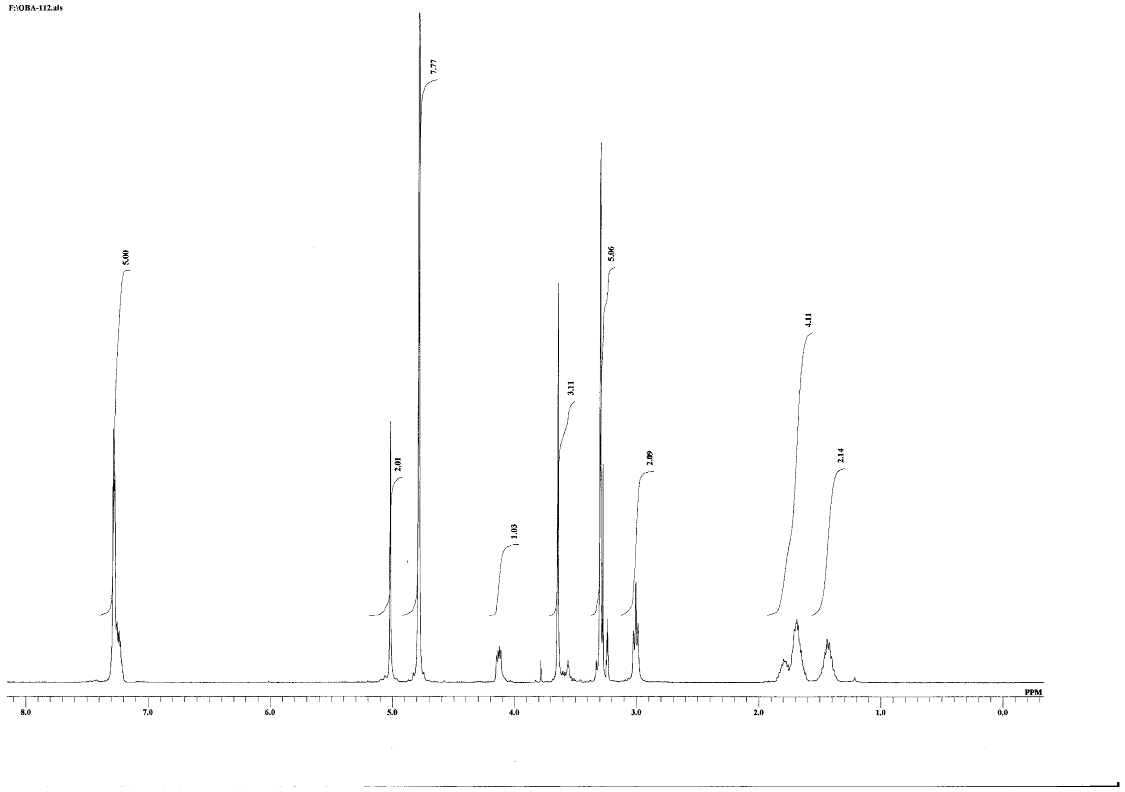

^{13}C NMR spectrum of **13**

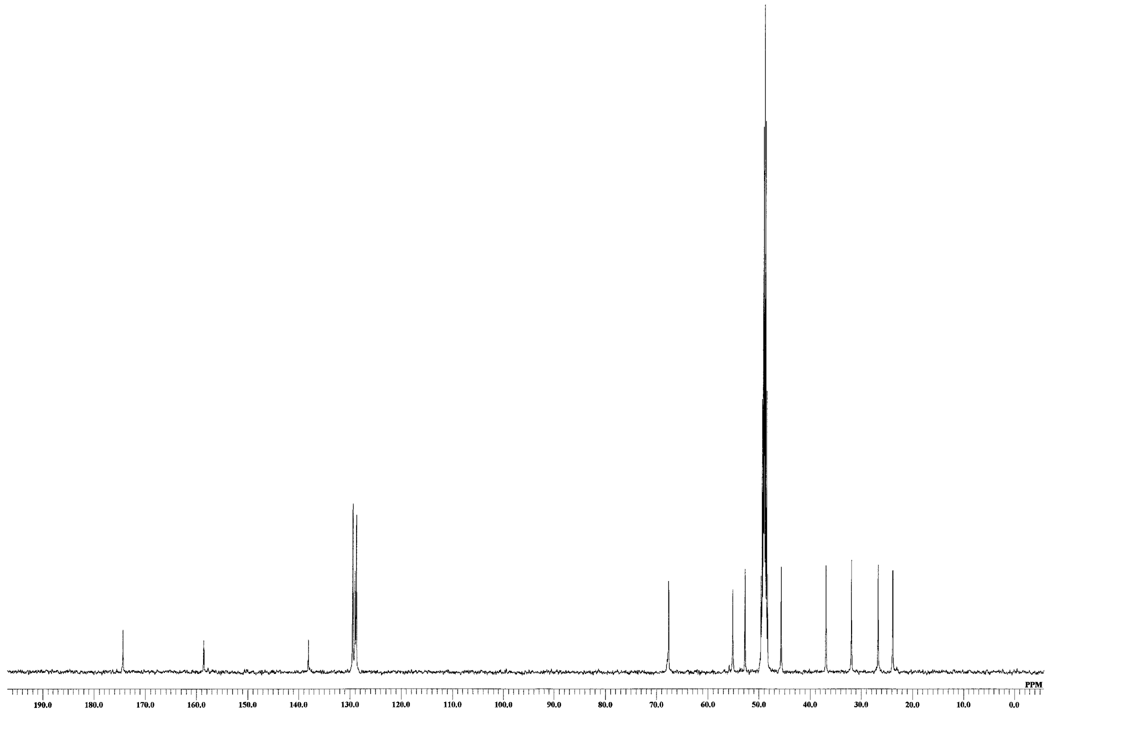

^{1}H NMR spectrum of **15**

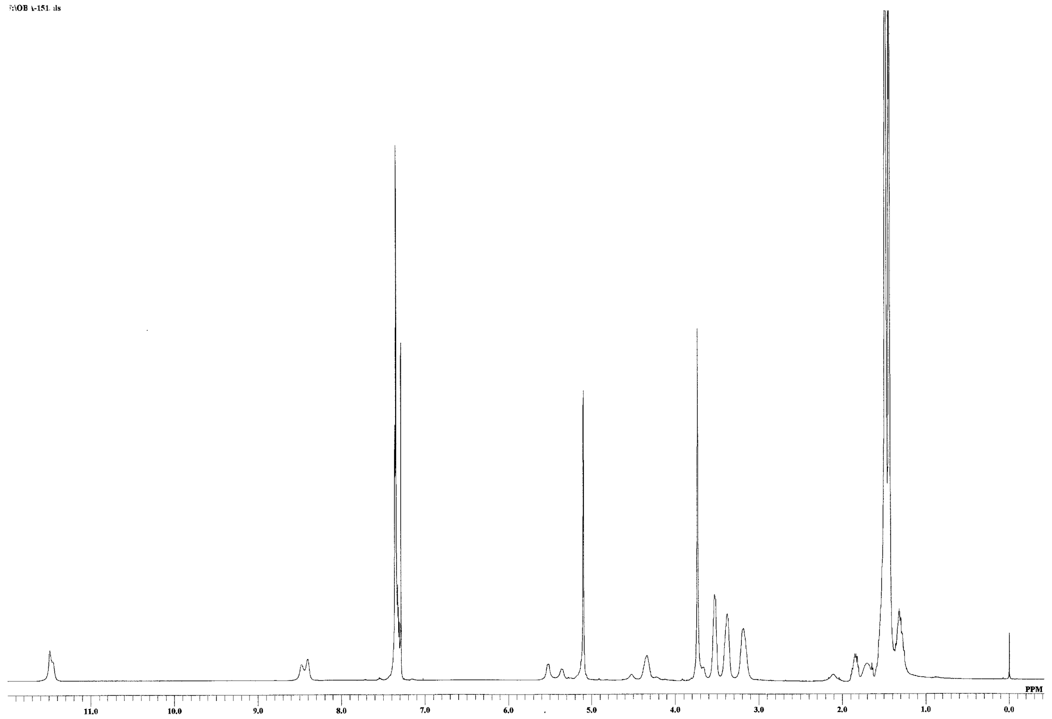

^{13}C NMR spectrum of **15**

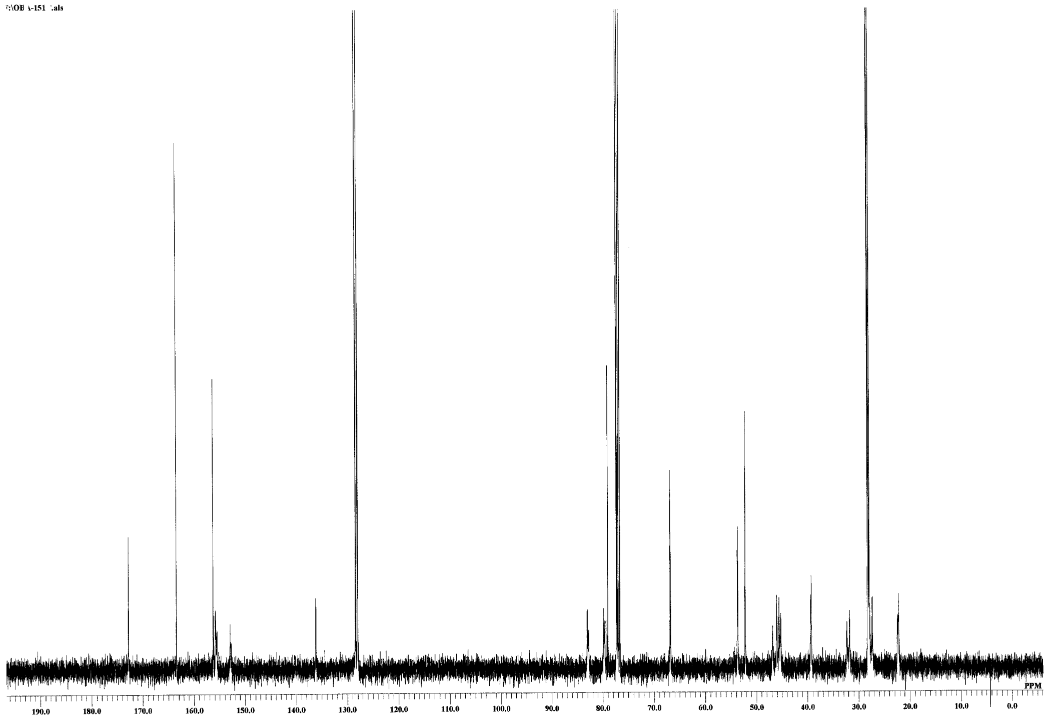

^{1}H NMR spectrum of **18**

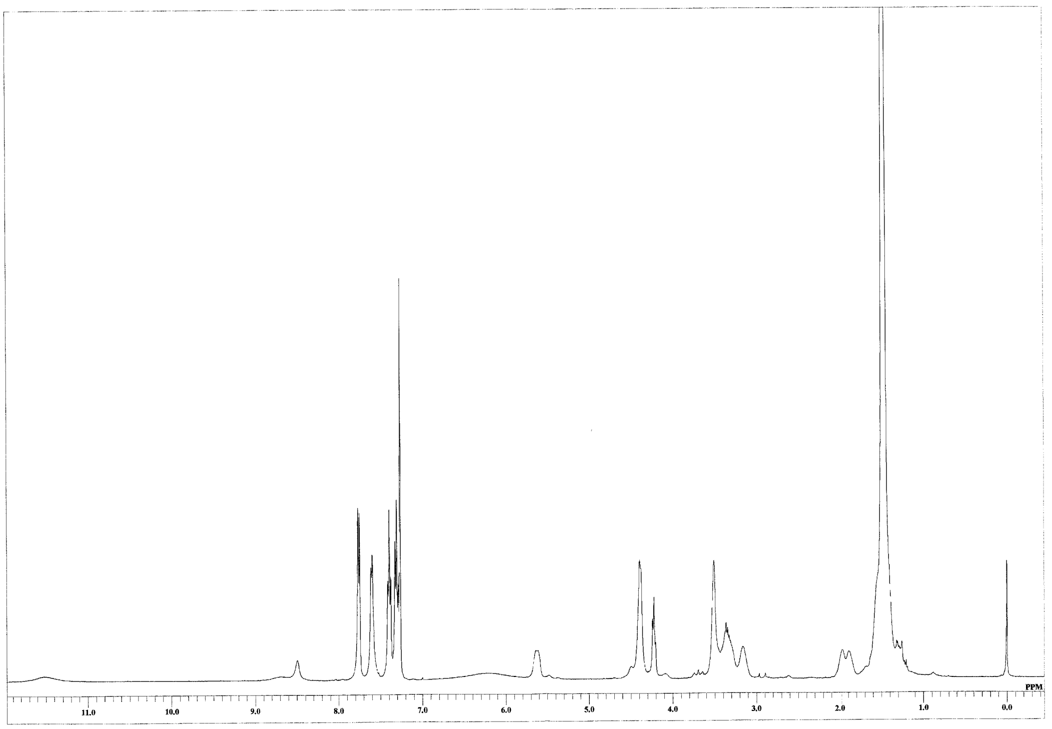

^{13}C NMR spectrum of **18**

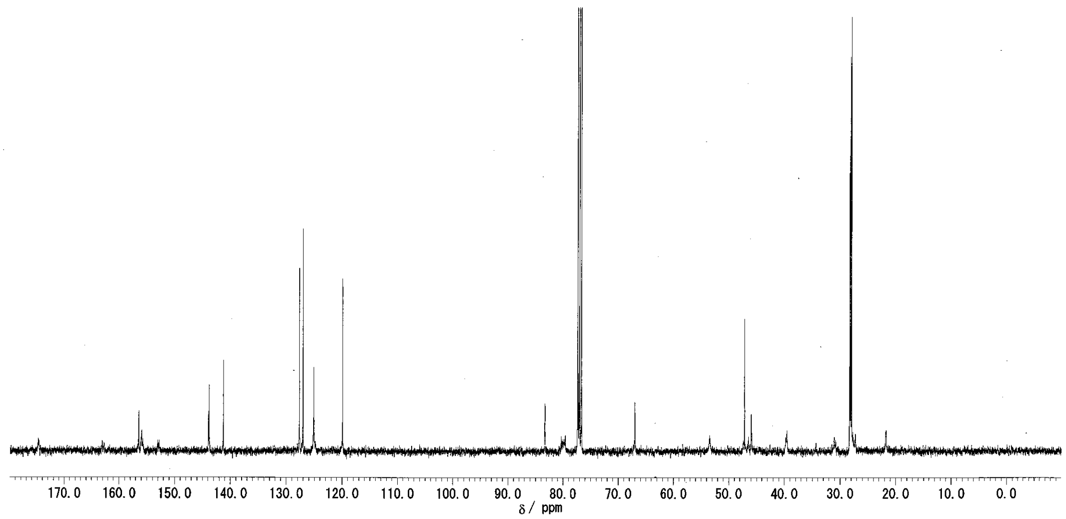

^{1}H NMR spectrum of **21**

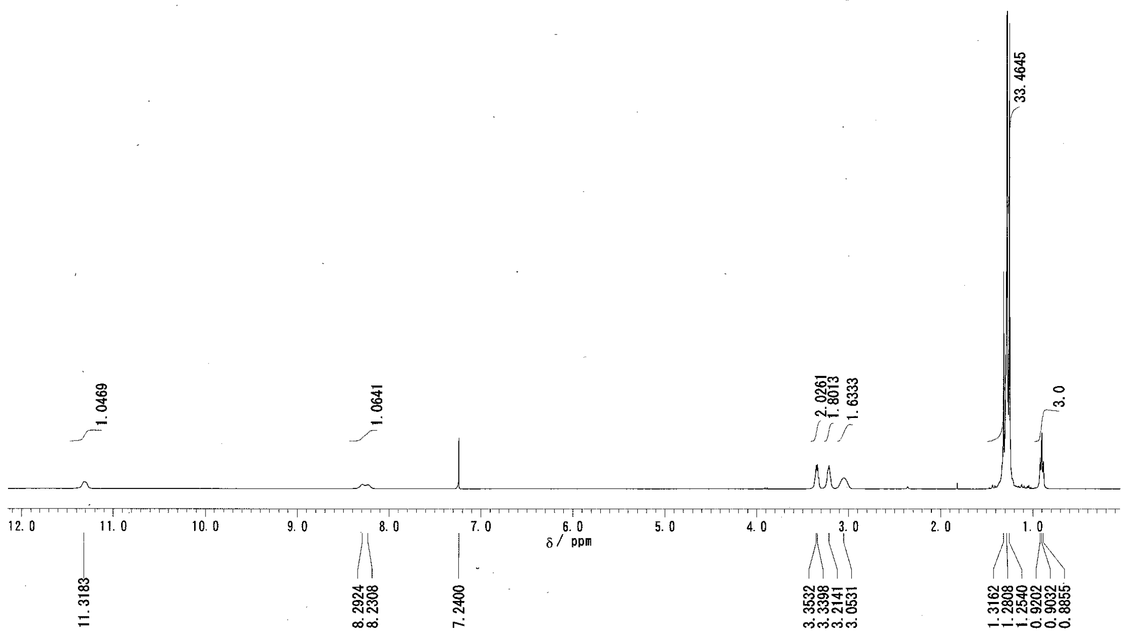

^{13}C NMR spectrum of **21**

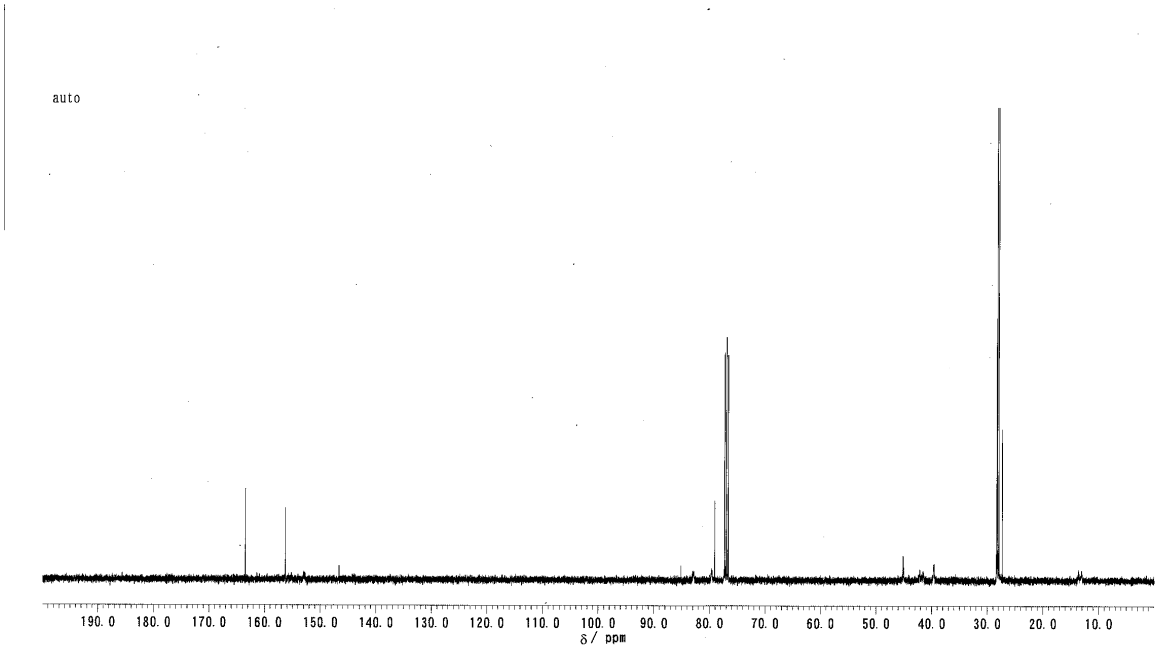

^{1}H NMR spectrum of **22**•2HCl

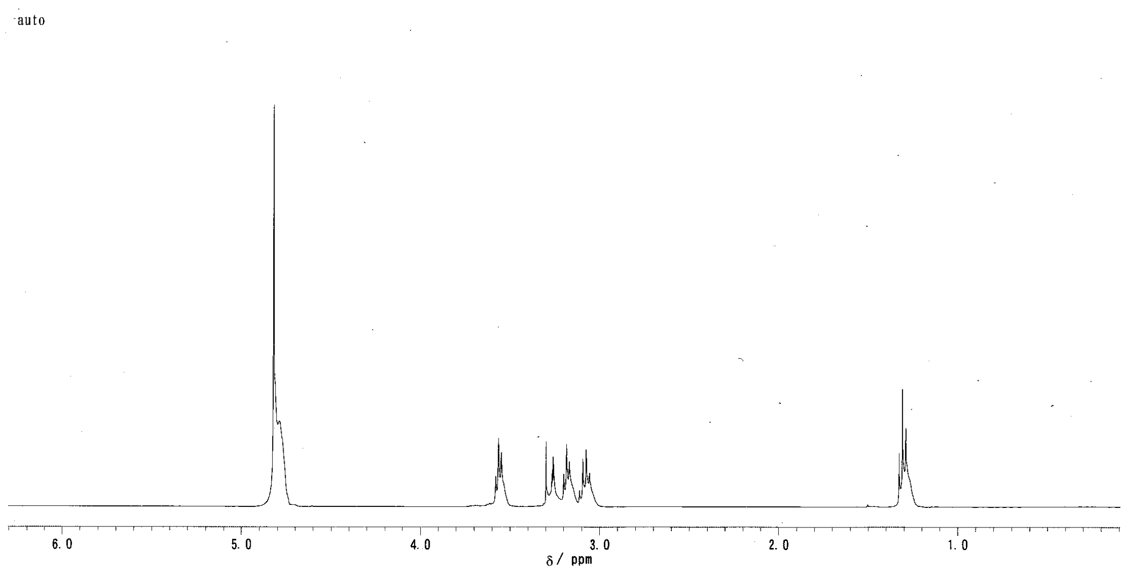

^{13}C NMR spectrum of **22**•2HCl

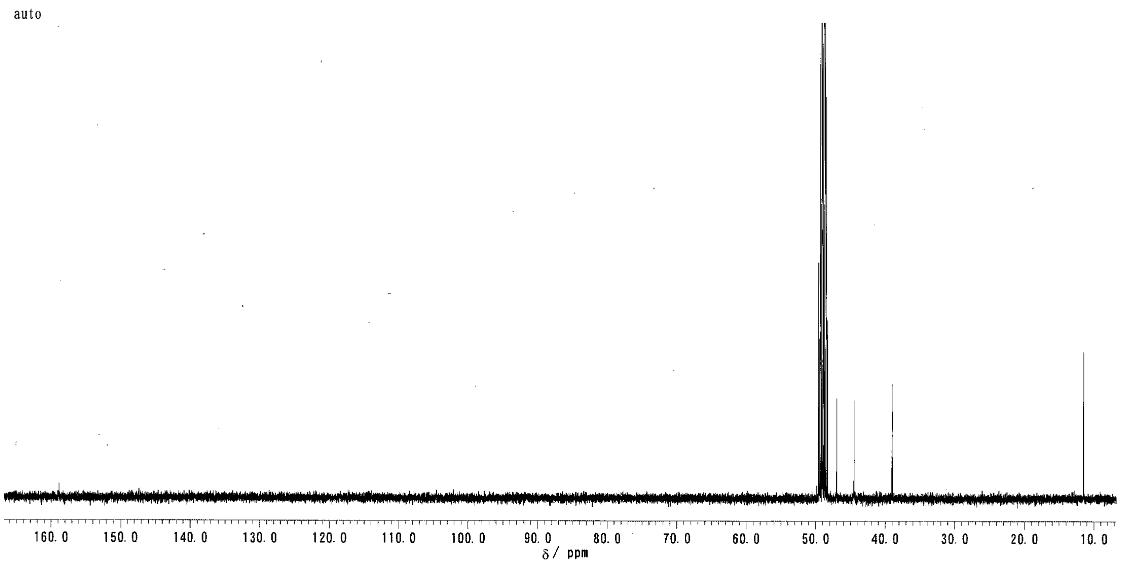

Reference

[S1] Feichtinger, K., Zapf, C., Sings, H. L., & Goodman, M. Diprotected triflylguanidines: A new class of guanidinylation reagents. *J. Org. Chem.* **63**, 3804–3805 (1998).

[S2] Yamashita, H. *et al*. Amphipathic short helix-stabilized peptides with cell-membrane penetrating ability. *Bioorg. Med. Chem.* **22**, 2403–2408 (2014).

[S3] Kato, T., Oba, M., Nishida, K., & Tanaka, M. Cell-penetrating helical peptides having L-arginines and five-membered ring α,α-disubstituted α-amino acids. *Bioconjugate Chem.* **25**, 1761−1768 (2014).
